# Effects of Different Pollens on Primary Metabolism and Lignin Biosynthesis in Pear

**DOI:** 10.3390/ijms19082273

**Published:** 2018-08-02

**Authors:** Shumei Li, Xueqiang Su, Muhammad Abdullah, Yanming Sun, Guohui Li, Xi Cheng, Yi Lin, Yongping Cai, Qing Jin

**Affiliations:** School of Life Science, Anhui Agricultural University, Hefei 230036, China; lishushumei@163.com (S.L.); 15710006@ahau.edu.cn (X.S.); abdullahpadana@hotmail.com (M.A.); 18225863536@163.com (Y.S.); zhuzhu3278@sina.com (G.L.); chengxi09@ahau.edu.cn (X.C.); linyi1957@126.com (Y.L.)

**Keywords:** ‘Dangshan Su’ pear, GC-MS, UPLC-MS/MS, different pollinations, metabolomics, lignin, stone cell

## Abstract

To investigate the effect of pollination on the fruit quality of ‘Dangshan Su’ pear, ‘Dangshan Su’ was fertilized by the pollen of ‘Wonhwang’ (*Pyrus pyrifolia* Nakai.) (DW) and ‘Jingbaili’ (*Pyrus ussuriensis* Maxim.) (DJ). The analysis of primary metabolites was achieved through untargeted metabolomics, and the quantitative analysis of intermediate metabolites of lignin synthesis was undertaken using targeted metabolomics. The untargeted metabolomics analysis was performed via gas chromatography-mass spectrometry (GC-MS). The targeted metabolomics analysis was performed using ultra-high-performance liquid chromatography-tandem mass spectrometry (UPLC-MS/MS) under the multiple reaction monitoring (MRM) mode. The results showed that the metabolite content was significantly different between DW and DJ. Compared with that in DJ, the sugar and amino acid content in DW was higher and the fatty acid content was lower at 47 days after pollination (DAPs), and the sugar, amino acid, and fatty acid content in DW was lower at 63 DAPs. The intermediate metabolites of lignin synthesis were analyzed using the orthogonal partial least squares discriminant analysis (OPLS-DA) model, and the differential metabolites at 47 DAPs were *p*-coumaric acid, ferulic acid, sinapaldehyde, coniferyl alcohol, and sinapyl alcohol. The differential significant metabolite at 63 DAPs was *p*-coumaric acid. At 47 DAPs and 63 DAPs, the *p*-coumaric acid level was significantly different, and the *p*-coumaric acid content was positively correlated with lignin synthesis. The pollination pollen affects the quality of ‘Dangshan Su’ pear fruit through regulation of the sugar, amino acid, and fatty acid content; at the same time, regulating the levels of intermediate metabolites of lignin synthesis, especially the *p*-coumaric acid content, to affect lignin synthesis ultimately affects the stone cell content and improves the quality of the pears.

## 1. Introduction

*Pyrus bretschneideri* cv. ‘Dangshan Su’ is a cultivar of *Pyrus bretschneideri* Rehd. Pollination can affect pear fruit quality, such as its true hardness and sugar, amino acid, and stone cell content [1,2,3,4]. During the ripening of pear fruit, a large number of substances are synthesized. Previous studies have focused on the metabolites related to taste, color, aroma, and nutrition in mature fruits. A qualitative and quantitative analysis of sugar, organic acids, amino acids, and fatty acids in different varieties of pear fruits showed that the composition of different pear fruit varieties varied greatly [5]. The phenylpropanoid pathway is the junction between secondary metabolism and primary metabolism, such as lignin synthesis. The phenylpropanoid pathway is derived from the shikimic acid pathway, and the shikimic acid pathway can be formed via phosphoenolpyruvate in the primary metabolic glycolytic pathway. In addition, the phenylpropanoid pathway is not only related to the biosynthesis of lignin and flavonoids but also related to other aromatic metabolites, such as coumarin, phenolic volatiles, and hydrolysable tannins [6,7]. Oikawa analyzed the metabolic profiles of pear fruits and determined the physiological effects of the metabolites in pear fruits [8]. However, there has been no report on the effects of pollination on the primary metabolites in ‘Dangshan Su’ pear fruit.

The lignin synthesis process has been basically elucidated [9], and the types of lignin contained in the pear fruit are guaiacyl lignin (G-lignin) and syringyl lignin (S-lignin) [10]. Lignin synthesis, translocation, and deposition are closely related to the development of stone cells. Pearlite cells are thick-walled tissue cells formed by the deposition of lignin on the parenchyma of parenchyma cells and secondary thickening of the primary wall. The pear fruit quality is related to the content of stone cells [11,12]. The effect of pollination on lignin synthesis in pear fruit can theoretically be a basis for improving pear fruit quality.

Previous studies have reported that pollination affects the expression of laccase gene microRNA in pear fruit [4], and the expression of peroxidase 47 (PER47), β-glucosidase (BGLU15), and laccase-4 (LAC4) [13], thus affecting lignin synthesis. This finding demonstrates that pollination with different pollens affects the synthesis of lignin in pear fruit on the levels of gene and protein expression. However, there has been no report regarding the effect of pollination on metabolites in ‘Dangshan Su’ pear fruit. In this study, the primary metabolites in ‘Dangshan Su’ fruit pollinated by ‘Wonhwang’ (DW) and ‘Jingbaili’ (DJ) at 47 and 64 days after pollination (DAPs) were detected, combined with quantitative detection of the lignin synthesis intermediate metabolites cinnamic acid, *p*-coumaric acid, caffeic acid, ferulic acid, sinapic acid, coniferaldehyde, sinapaldehyde, coniferyl alcohol, and sinapyl alcohol to investigate the effects of pollination on primary metabolism and lignin synthesis in ‘Dangshan Su’ pear fruit and to improve the pear fruit quality.

According to the previous experimental results of this research group, lignin mainly forms between 15 days and 63 days after flowering [2,4,11,12]. The content of lignin and stone cells in DW and DJ has been detected. At 47 DAPs, the lignin content reaches a peak, which is the key period of lignin formation. At 63 DAPs, there are significant differences in the lignin content and stone cell content in pear fruits [13]. Therefore, fruit at 47 and 63 DAPs was selected for metabolomics analyses.

## 2. Results

### 2.1. Un-Targeted Metabolomics Analysis of Primary Metabolites in ‘Dangshan Su’ Pear Fruit with Different Pollens

**Figure 1:**
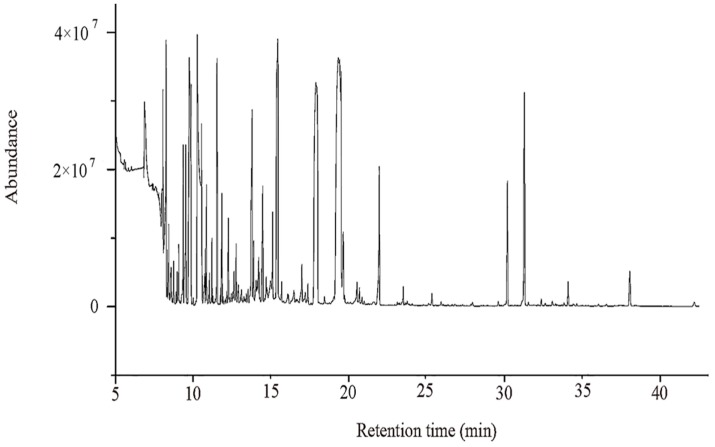
Overlays of total ion chromatograms (TICs) from GC-MS analysis of metabolites in pear fruit.

**Figure 2:**
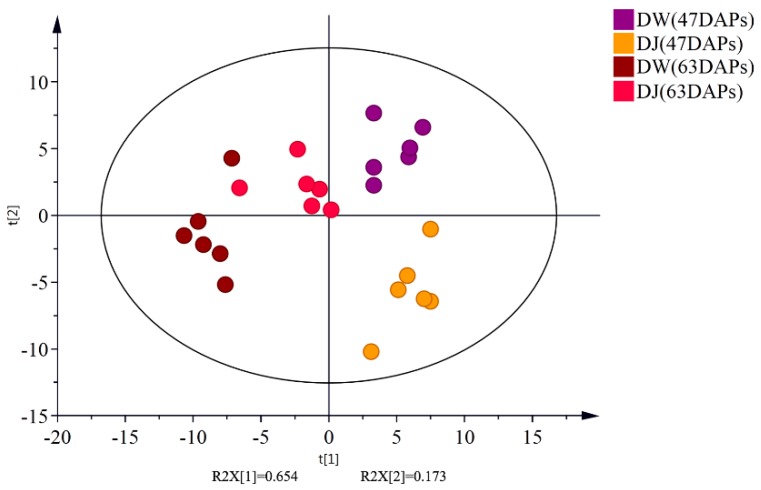
Principle component analysis (PCA) of the primary metabolites derived from GC-MS data.

The total ion chromatograms (TICs) from GC-MS analyses of DW and DJ are shown in Figure 1: 711 substance peaks were detected, 218 compounds were detected, and 202 metabolites were appraised, including sugars, organic acids, amino acids, nucleosides, nucleotides, and aromatic substances. At 47 DAPs, a total of 173 and 164 metabolites were identified in DW and DJ, respectively; at 63 DAPs, a total 163 and 170 metabolites were identified in DW and DJ. To reduce the differences caused by each sampling, all peak signal strengths (peak areas) were normalized. A multivariate statistical analysis was performed on the normalized data. The first principal component contains 18.4% variables, and the second principal component contains 10.3% variables. A good distinction can be made between DW and DJ (Figure 2).

#### 2.1.1. Effect of Pollination on Primary Metabolites in Dangshan Pear Fruit

**Figure 3:**
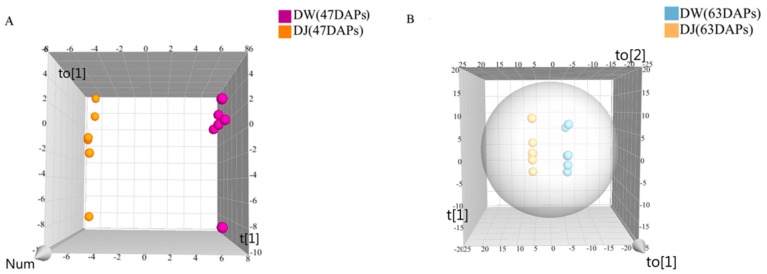
Orthogonal partial least squares-discriminant analysis (OPLS-DA) of metabolites derived from GC-MS. (**A**): 47 days after pollination (DAPs); (**B**): 63 DAPs.

The orthogonal partial least squares-discriminant analysis (OPLS-DA) model was applied to the GC-MS data (Figure 3). As shown in Figure 3A, the R^2^Y, and Q^2^ values were 0.999 and 0.886, respectively. The R^2^Y and Q^2^ values presented in Figure 3B were 1 and 0.8446, respectively. This result showed that the OPLS-DA model was stable and reliable. The OPLS-DA model was used according to a variable influence on projection (VIP) values and *p*-values to select differential metabolites. Metabolites with VIP > 1 and *p* < 0.05 were select as differential metabolites. At 47 DAPs, there were 51 differential metabolites, and 8 differential metabolites were only detected in DW; the levels of 26 differential metabolites were higher in DW, and 17 differential metabolite levels were higher in DJ (Table 1). At 63 DAPs, there were 34 differential metabolites; 1 differential metabolite was only detected in DW, and 7 differential metabolites were only detected in DJ. The levels of 5 differential metabolites were higher in DW, and those of 27 differential metabolites were higher in DJ (Table 2).

#### 2.1.2. Effects of Pollination on Carbohydrate Metabolism and Amino Acid Metabolism in Dangshan Pear Fruit

**Figure 4:**
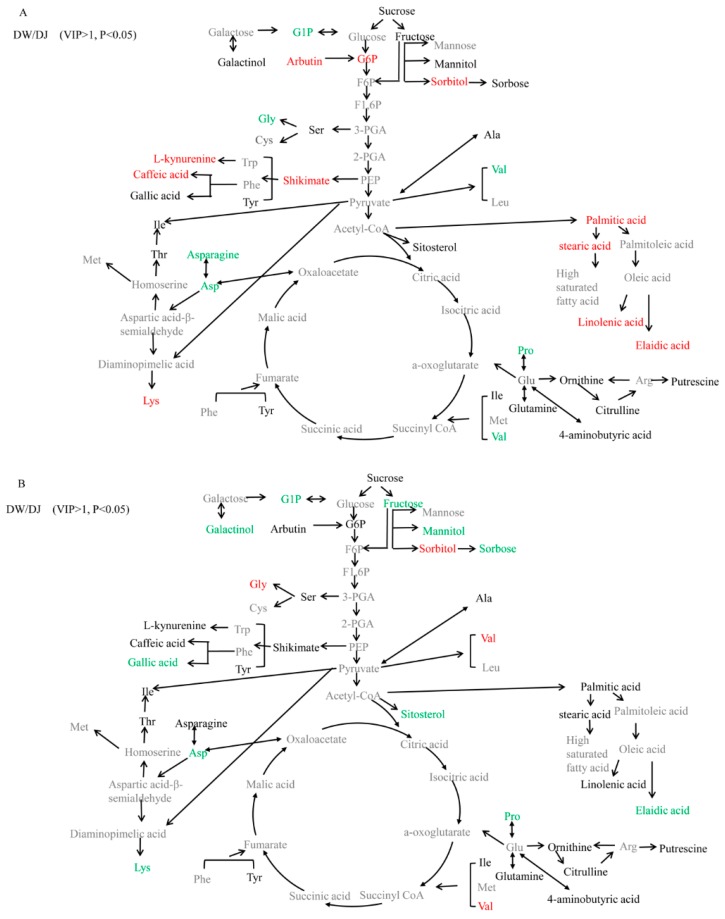
Changes in metabolites in DW and DJ shown in a metabolic diagram. (**A**) The changes at 47 DAPs; (**B**) The changes at 63 DAPs. The metabolites with red characters were detectable and exhibited higher content in DW than in DJ. The metabolites with labelled in green were detectable but with lower content in DW than in DJ. The metabolites with black characters were detectable. The metabolites with grey characters were undetectable. G1P, glucose-1-phosphate; G6P, glucose-6-phosphate; F6P, fructose-6-phosphate; F1,6P, fructose-1,6-diphosphate; 3-PGA, 3-phosphoglyceraldehyde; 2-PGA, 2-phosphoglyceraldehyde; PEP, phosphoenolpyruvate; Gly, glycine; Cys, cysteine; Ser, serine; Trp, tryptophan; Phe, phenylalanine; Tyr, tyrosine; Ile, isoleucine; Thr, threonine; Met, methionine; Asp, aspartate; Lys, lysine; Ala, alanine; Val, valine; Leu, leucine; Glu, glutamate; Pro, proline; Arg, arginine.

To clarify changes in the metabolic pathways of DW and DJ, differential metabolites were marked on a metabolic diagram (Figure 4). At 47 DAPs (Figure 4A), the content of glucose-6-phosphate (1.850 times), arbutin (1.705 times), sorbitol (1.583 times), shikimic acid (1.446 times), caffeic acid (3.383 times), palmitic acid (1.719 times), stearic acid (1.890 times), linolenic acid (1.930 times), and elaidic acid (1.568 times) was higher in DW; l-kynurenine and lysine were only detected in DW. The glucose-1-phosphate (0.599 times), glycine (0.536 times), valine (0.394 times), aspartic acid (0.385 times) and proline (0.359 times) content was lower in DW, but asparagine was only detected in DJ.

At 63 DAPs (Figure 4B), the sorbitol (2.293 times), glycine (1.396 times) and valine (5.355 times) content was higher in DW; the fructose (0.615 times), galactose glucoside (0.579 times), mannitol (0.478 times), sitosterol (0.702 times), aspartic acid (0.557 times), proline (0.472 times), oleic acid (0.677 times) and gallic acid (0.605 times) levels were lower in DW; and sorbose and lysine were only detected in DJ.

### 2.2. Effects of Pollination on Intermediate Metabolites of Lignin Synthesis in Dangshan Pear Fruit

#### 2.2.1. Effects of Pollination on the Content of Intermediate Metabolites in Lignin Synthesis of Dangshan Pear Fruit

By comparing the retention time (Figure S1) and characteristic ion pairs (Figure S2) between the standards and samples, 7 lignin synthesis metabolites were detected. The multiple reaction monitoring (MRM) scanning chromatogram of a standard and experimental sample is shown in Figures S3 and S4, respectively.

**Figure 5:**
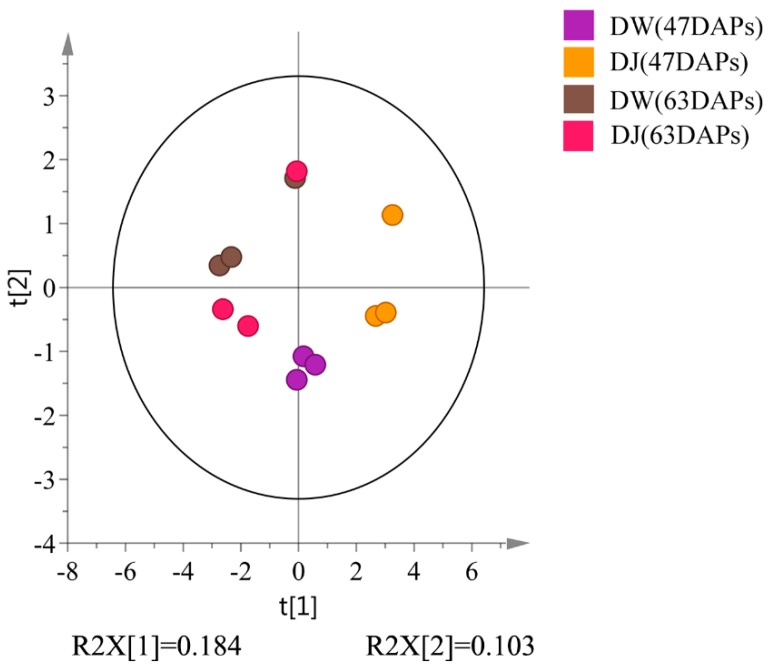
Principle component analysis (PCA) of the lignin metabolites derived from LC-MS.

The PCA scores of samples were a good fit (R^2^ = 0.827) and exhibited good predictive value (Q^2^ = 0.415) in the PCA scoring diagram (Figure 5). The first principal component contains 65.4% variables, and the second principal component contains 17.3% variables. The PCA scores showed that the distribution of the samples was roughly the same and that there was no sample out of the scoring chart Hotelling T2 95% confidence interval; thus, it can be used for subsequent analysis [14].

**Figure 6:**
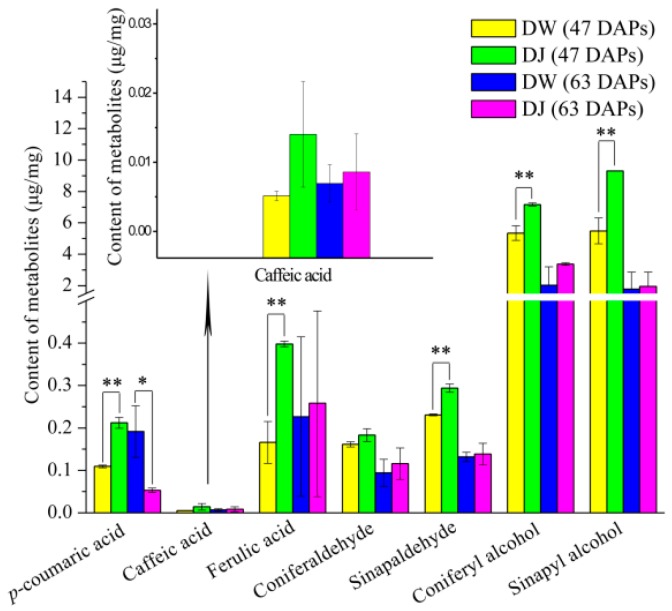
The lignin synthesis metabolite content in DW and DJ. Note: * represents a significant difference at the 0.05 leve (two-tailed Student’s *t*-test); ** represents a significant difference at the 0.01 leve (two-tailed Student’s *t*-test). The error bars represent standard deviations [20].

The experimental data of targeted analysis with a two-tailed Student’s *t*-test. As seen in Figure 6, the *p*-coumaric acid, caffeic acid, ferulic acid, coniferaldehyde, sinapaldehyde, coniferyl alcohol, and sinapyl alcohol content in DW is lower than that in DJ at 47 DAPs. This result was consistent with the content of lignin and stone cells at 47 DAPs. The *p*-coumaric acid, ferulic acid, sinapaldehyde, coniferyl alcohol and sinapyl alcohol content was statistically different. At 63 DAPs, the *p*-coumaric acid content in DW was significantly higher than that in DJ, approximately 3.62 times higher. The lignin content could increase when the *p*-coumaric acid content was increased [15,16]. This result was consistent with the observation that the lignin content was higher in DW. The results showed that different pollens could affect the lignin content by regulating the intermediate metabolite content in lignin biosynthesis.

**Figure 7:**
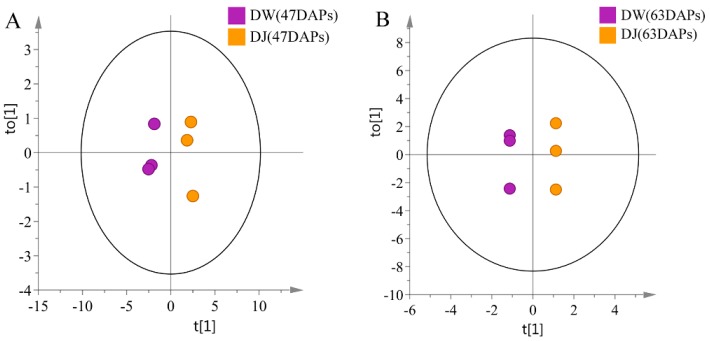
Orthogonal partial least squares-discriminant analysis (OPLS-DA) of lignin metabolites derived from LC-MS. (**A**): 47 DAPs; (**B**): 63 DAPs.

Through OPLS-DA analysis of the targeted metabonomics, it was found that the model has good quality at 47 DAPs (R^2^Y = 0.983, Q^2^ = 0.983) and 63 DAPs (R^2^Y = 1, Q^2^ = 0.982) [17]. It can be seen from the OPLS-DA scores (Figure 7) that the pear fruit can be clearly distinguished between DW and DJ. VIP > 1 and *p* < 0.05 were used as the criteria for selecting differential metabolites [18,19]. The differential metabolites at 47 DAPs were *p*-coumaric acid, ferulic acid, sinapaldehyde, coniferyl alcohol and sinapyl alcohol. At 63 DAPs, the differential metabolite was *p*-coumaric acid (Table 4).

#### 2.2.2. Correlation Analysis of Intermediate Metabolites in Lignin Synthesis in Pear Fruit

**Figure 8:**
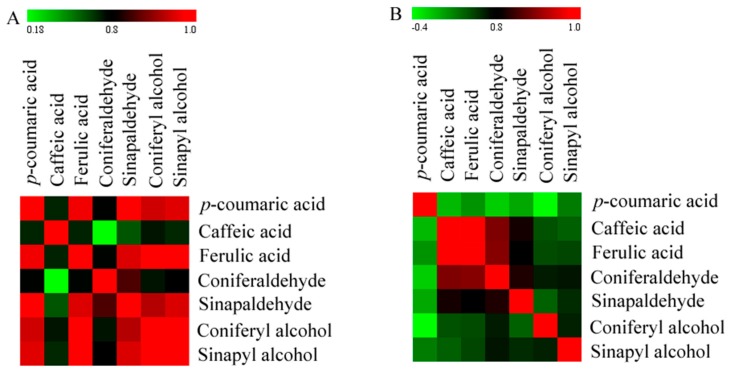
Heat map representing the correlations of lignin intermediate metabolites in pear fruit. (**A**): 47 DAPs; (**B**): 63 DAPs.

To study the effects of pollination on lignin synthesis in pear fruit, a Pearson analysis of correlations between lignin synthesis intermediate metabolites was performed (Figure 8). The results showed that at 47 DAPs most of intermediate metabolites of lignin synthesis were significantly positively correlated (*r* > 0.08, *p* < 0.05), and a few metabolites were not correlated. At 63 DAPs, most of intermediate metabolites of lignin synthesis were not correlated, and metabolites showed significant positive correlation.

**Figure 9:**
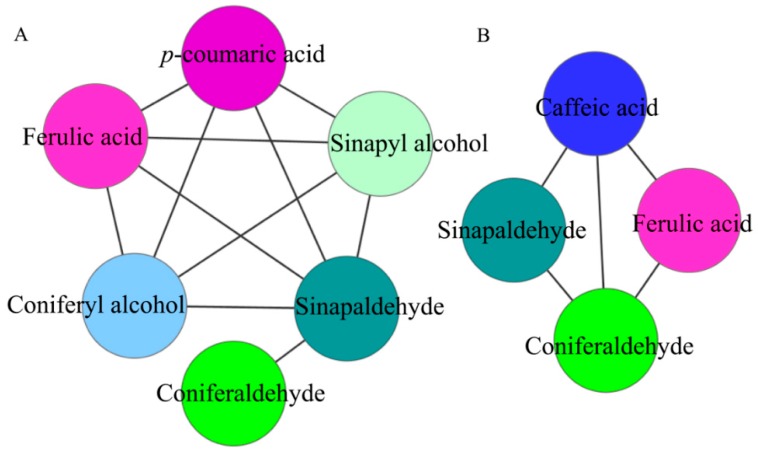
Correlation network of significantly related lignin synthesis intermediate metabolites in pear fruit. (**A**): 47 DAPs; (**B**): 63 DAPs. Node colors represent different types of metabolites.

A correlation network analysis was performed on the intermediate metabolites of lignin synthesis to determine significantly positive correlations (*r* > 0.8, *p* < 0.05) (Figure 9). At 47 DAPs, 11 pairs of metabolites exhibited significantly positive correlation. *p*-Coumaric acid and ferulic acid (*r* = 0.963, *p* = 2.011 × 10^−3^), sinapaldehyde (*r* = 0.986, *p* = 2.905 × 10^−3^), coniferyl alcohol (*r* = 0.938, *p* = 5.461 × 10^−3^), and sinapyl alcohol (*r* = 0.952, *p* = 3.447 × 10^−3^) had a significantly positive correlation. Ferulic acid and sinapaldehyde (*r* = 0.949, *p* = 3.868 × 10^−3^), coniferyl alcohol (*r* = 0.994, *p* = 5.057 × 10^−4^), and sinapaldehyde (*r* = 0.972, *p* = 1.16) had a significantly positive correlation. Sinapaldehyde and coniferaldehyde (*r* = 0.853, *p* = 3.071 × 10^−2^), coniferyl alcohol (*r* = 0.921, *p* = 9.213 × 10^−3^), and sinapyl alcohol (*r* = 0.948, *p* = 4.044 × 10^−3^) had a significantly positive correlation. Coniferyl alcohol and sinapyl alcohol (*r* = 0.976, *p* = 8.360 × 10^−4^) had a significantly positive correlation.

At 63 DAPs, 5 pairs of metabolites were positively correlated. Caffeic acid was positively correlated with ferulic acid (*r* = 0.976, *p* = 8.878 × 10^−4^), coniferaldehyde (*r* = 0.886, *p* = 1.872 × 10^−2^), and sinapaldehyde (*r* = 0.821, *p* = 4.517 × 10^−2^). Ferulic acid was positively correlated with coniferaldehyde (*r* = 0.891, *p* = 1.716 × 10^−2^). There was a significant positive correlation between coniferaldehyde and sinapaldehyde (*r* = 0.827, *p* = 4.221 × 10^−2^).

## 3. Discussion and Conclusions

Metabolomic analysis does not rely on transcriptomics, proteomics, or genomic sequences and has a wide range of applications. This analysis is one of the most effective methods for understanding the mechanisms of metabolic regulation [21,22]. In this experiment, GC-MS and LC-ESI-MS/MS methods were used for detection. Compared with NMR spectroscopy, these techniques have more extensive coverage and more complex univariate and multivariate data analysis methods [23]. In addition, LC-ESI-MS/MS has the advantages of high precision, high sensitivity, and high output [24]. In this study, LC-ESI-MS/MS data were used to rapidly and sensitively characterize and quantify 7 metabolites in pear fruit via MRM [25].

Target detection of pear fruit primary metabolites with GC-MS, PCA, and OPLS-DA showed that DW and DJ can clearly be distinguished. Different pollens have a great influence on metabolites. Sugar, amino acids, and fatty acids are associated with pear fruit quality [5]. At 47 DAPs, the sugar and amino acid content is relatively higher in DW, and the fatty acid content is relatively lower. At 63 DAPs, the sugar, amino acid, and fatty acid content in DW is lower than that in DJ. It can be speculated that different pollens could affect the quality of pear fruit.

At 47 DAPs, the KEGG pathways are enriched by differential metabolites, including phenylalanine, tyrosine and tryptophan biosynthesis (Table 3), and the synthesis of lignin is initiated by the deamination of phenylalanine to form cinnamic acid [26]. Therefore, it can be inferred that pollination affects the synthesis of phenylalanine in pear fruit and regulates the synthesis of lignin.

Through the targeted metabolomics analyses of DW and DJ, along with the OPLS-DA, at 47 DAPs, the differential metabolites identified were *p*-coumaric acid, ferulic acid, sinapaldehyde, coniferyl alcohol and sinapyl alcohol. At 63 DAPs, the differential metabolite was *p*-coumaric acid. Therefore, it is speculated that different pollens affect the formation of lignin synthesis intermediate metabolites in pear fruit and then regulate the formation of lignin. At 47 DAPs, different pollens had a great influence on the formation of lignin.

The differential metabolites included *p*-coumaric acid both at 47 DAPs and 63 DAPs. The *p*-coumaric acid content in DW was 0.52 times and 3.62 times higher than that in DJ at 47 DAPs and 63 DAPs, respectively; at the same time, the lignin content was 0.47 times and 2.55 times higher, respectively. It can be seen that when the lignin content in pear fruit is high, the *p*-coumaric acid content is also high. Through multiple regression analysis, it was found that *p*-coumaric acid and lignin were significantly positively correlated (R^2^ = 0.733, *p* < 0.01). The *p*-coumaric acid content could affect the synthesis of lignin [27,28]. The synthesis of pear lignin mainly initiated from *p*-coumaric acid, consistent with the results of Cai [2]. *p*-Coumaric acid is a precursor to the synthesis of lignin in pear fruit, which is closely related to the synthesis of lignin and the formation of stone cells [29].

At 47 DAPs, 11 pairs of lignin synthesis intermediate metabolites were significantly positively correlated. At 63 DAPs, 5 pairs of lignin synthesis intermediate metabolites were significantly positively correlated. It can be seen that 47 DAPs is a critical period for lignin formation in pear fruit. This result is consistent with previous results [13].

## 4. Materials and Methods

### 4.1. Materials

Fruits were obtained from 50-year-old pear trees grown in Dangshan, Anhui, China. In April, twenty robust and healthy ‘Dangshan Su’ managed in a consistent manner were selected as mother trees. The ‘Wonhwang’ (*Pyrus pyrifolia* Nakai.) and ‘Jingbaili’ (*Pyrus ussuriensis* Maxim.) were selected as father trees, and the pollen was collected from buds with similar developmental stages and sizes. Buds on the short branches of ‘Dangshan Su’ with similar developmental stages and sizes were selected from the mid-crown area on the south side of each tree, and the stamen was removed and fertilized with pollen from *P. pyrifolia* cv. ‘Wonhwang’ (DW) and *P. ussuriensis* cv. ‘Jingbaili’ (DJ). Two fruits were kept for each short branch and covered with bags for seven days after pollination. Fruits were collected at 47 and 63 DAPs. Twenty fruits with relatively uniform size were collected at each time point, refrigerated, and transferred to the laboratory for further study. The standard samples of cinnamic acid, *p*-coumaric acid, caffeic acid, ferulic acid, sinapic acid, coniferaldehyde, sinapaldehyde, coniferyl alcohol and sinapyl alcohol were purchased from Sigma-Aldrich (St. Louis, MO, USA) (purity > 99%).

Methanol (HPLC grade) was purchased from TEDIA (Fairfield, OH, USA). Pyridine was obtained from Ehrenstorfer (Augsburg, Germany). *N*,*O*-bis (trimethylsilyl)-trifluoroacetamide (BSTFA) containing 1% trimethylchlorosilane (TMCS) was purchased from SUPELCO (Bellefonte, PA, USA). Water was obtained from Wahaha Group Co., Ltd. (Hangzhou, China).

### 4.2. Preparation and LC-MS Detection of Lignin Synthesis Intermediate Metabolite Standard Solution

According to the difference in mass spectrometry response signals, the standards were weighed and dissolved in chromatographic-grade methanol solution to obtain standard solutions of different concentrations. Concentrations of *p*-coumaric acid, ferulic acid, coniferaldehyde, coniferyl alcohol, and sinapyl alcohol: 10, 30, 40, 80 and 500 ng/mL; cinnamic acid concentrations: 30, 40, 80, 500 and 1000 ng/mL; caffeic acid concentrations: 5, 10, 30, 40 and 80 ng/mL; sinapic acid concentrations: 5, 30, 40 and 80 ng/mL; and sinapaldehyde concentrations: 5, 10, 40, 80 and 500 ng/mL.

Standard solutions were analysed using ultra-high-performance liquid chromatography coupled with tandem mass spectrometry (UHPLC-MS/MS). The MS detection was performed on a Qtrap 6500 MS (AB Sciex, Framingham, MA, USA) equipped with an ESI source operating in negative-ion mode. Quantification of 9 metabolites was carried out in the MRM mode [30,31]. At the same time, optimization to voltage (DP), cluster collision (CE) and mass spectrum parameters, such as the optimal conditions for 9 types of compound ion pairs for rapid screening, was performed. The optimized mass spectrometry conditions were as follows: the gas curtain gas 30; collision gas 8; ion spray voltage −4500 V, temperature 550; ion source gas 1:55; and gas 2:55. The MRM detection parameters of 9 compounds are shown in Table 5, and the corresponding MRM changes were measured to determine the retention time of each metabolite [32,33].

The eluents were as follows: A, water with 0.1% formic acid; and B, acetonitrile with 0.1% formic acid. The gradient program was as follows: 10–10% B (3 min), 10–95% B (10 min), 95–95% B (2 min), 95–10% B (0.1 min), and 10–10% B (2.9 min) at a constant flow of 0.2 mL/min. The standard curves and retention times were obtained by testing of the lignin synthesis intermediate metabolite standards (Table 6).

### 4.3. Extraction and Derivation of Primary Metabolites from Pear and Analysis by GC-MS

According to Roessner [34] and other methods, the extraction and derivation of primary metabolites in pears were performed as follows:

(1) A 100 mg sample frozen in liquid nitrogen was placed in a 2 mL centrifuge tube, and 1.4 mL of methanol (−20 °C precooled, chromatography grade) was added. The sample was ground with a ball mill and then vortexed for 1 min.
(2) The centrifuge tube was sealed with parafilm and placed in an ultrasonic cleaner at 40 °C for 30 min.
(3) After the end of the ultrasonication, the liquid and the residue was transferred from the 2 mL centrifuge tube to a 10 mL centrifuge tube, and 1.4 mL of purified water (precooled at 4 °C) was added. The sample was then centrifuged at 8000 rpm/min for 15 min.
(4) Then, 100 μL of the upper methanol/water mixture supernatant was placed in a glass flask, and the sample was dried with nitrogen and derivatized. First, 60 μL of hydroxylamine hydrochloride solution (hydroxylamine hydrochloride dissolved in pyridine, 20 mg/mL) was added, and then, the sample was sealed with parafilm, vortexed for 30 s, and placed in an oven at 37 °C for 120 min. Subsequently, 60 μL of derivatizing reagent (99% BSTFA + 1%TMCS) was added, and the flask was sealed and incubated at 37 °C for 90 min. After centrifugation at 12,000 rpm for 4 min, the supernatant was taken in the inner cannula, and the sample was tested. All samples were replicated six times. The test conditions were as follows:

Extracts of pear fruits were subjected to metabolic profiling analysis using an Agilent 7890B-5975C GC-MS system. Chromatographic separation was performed with a DB-5 MS fused-silica capillary column (30 m × 0.25 mm × 0.25 μm, Agilent Technologies, Santa Clara, CA, USA). The splitless injection volume was 1 µL. The column temperature was held at 40 °C for 4 min, increased to 190 °C at a rate of 15 °C/min, increased to 200 °C at a rate of 4 °C/min for 3 min, increased to 240 °C at a rate of 10 °C/min for 5 min, and then increased to 280 °C at a rate of 10 °C/min for 10 min. The carrier gas was helium (99.999%, Hefei Junjun Experimental Materials Co., Ltd., Hefei, China), and the flow rate was maintained at a constant linear velocity of 1.0 mL/min. The electron ionization source voltage was 70 eV with an electron impact (EI) ionization mass spectrometric detector (MSD). The ion source temperature was 230 °C, and the interface temperature was 280 °C. Data acquisition started at 5.0 min, with a mass range of 35–780 *m*/*z*.

### 4.4. Extraction and LC-MS Detection of Intermediate Metabolites from Pyrus Lignin

(1) Extraction of phenolic acid material: 100 mg of the samples was taken and frozen in liquid nitrogen. According to the material liquid volume, 80% ethanol solution was added at a 1:15 ratio, and the sample was ground with a ball mill, ultrasonicated for 40 min at 40 °C, and centrifuged to obtain the supernatant. The process was repeated for each group three times.
(2) Phenolic alcohol extract and phenolic substances: 100 mg of each sample was frozen in liquid nitrogen. According to the material liquid volume, 90% ethanol solution was added at a 1:15 ratio, and the sample was ground with a ball mill, ultrasonicated for 30 min at 70 °C, and centrifuged to obtain the supernatant. The process was repeated for each group three times.

After combination of the two supernatants, the nitrogen was blown dry, and 100 μL of acetonitrile was added. The sample was centrifuged at 12,000 rpm for 10 min, and the supernatant was removed. UPLC-ESI-MS/MS was used for qualitative and quantitative analysis of the metabolites of lignin synthesis.

### 4.5. Data Processing and Analysis

To pass the raw GC/MS data through ChemStation (version e. 02.02.1431, Agilent, Santa Clara CA, USA) analysis software, they must be converted into a common format (CDF format) by importing ChromaTOF (version 4.34, LECO, St Joseph, MI, USA) preprocessing software, including extraction, peak to noise, and deconvolution functions. The NIST and Fiehn databases were used for qualitative metabolite analysis and alignment, and finally, the peak data matrix was derived.

LC-MS/MS data were analyzed with Analyst 1.6.2 software (AB Sciex, Framingham, MA, USA) using the default parameters for automatic identification of the various MRM changes and integrals. In addition, an auxiliary artificial check was performed by examining retention times and characteristic ions compared with those of the standards to determine the detected compounds. The linear regression standard curve was plotted with the concentration of the analyte as the vertical coordinate. The mass spectra peak area of each metabolite in the sample was substituted into the linear equation of the standards, and the metabolite content in the samples was obtained.

SIMCA-P (version 14.0, Umetrics, Umea, Sweden) was imported to adopt unsupervised principal component analysis (PCA) of the GC-MS matrix in the numerical test results data and the lignin synthesis intermediate metabolites content to examine differences between the population distribution in the samples and the stability of the whole analysis process. Then, supervised orthogonal partial least squares-discriminant analysis (OPLS-DA) was used to differentiate between the metabolic profile of the general differences between groups and to find the differences between groups of metabolites. The differential metabolites were selected on the basis of the combination of the statistically significant threshold of variable influence on projection (VIP) values obtained from the OPLS-DA model and the *p*-value from a two-tailed Student’s *t*-test of the normalized peak area. The VIP value means variables have influence on classification [35]. Through this analysis, whether compounds in different groups significantly contributed (VIP > 1, *p* < 0.05) was determined [17,36]. We mapped the differential metabolites to the KEGG database (http://www.kegg.jp/kegg/pathway.html), which could be enriched to the KEGG pathway and confirmed the relationship between metabolite-metabolite correlations. SPSS software (IBM, Armonk, New York, USA) was also used for the statistical analyses, including correlations and significance analyses.

## Figures and Tables

**Table 1:** Differential metabolites at 47 DAPs in Wonhwang’ (DW) and ‘Jingbaili’ (DJ).

Metabolite	VIP	*p*-Value	Fold Change
3-hydroxypyruvate	1.959	2.760 × 10^−19^	+
l-kynurenine	1.958	4.438 × 10^−16^	+
*S*-carboxymethylcysteine	1.875	2.322 × 10^−6^	3.374
aspartic acid	1.849	4.036 × 10^−6^	0.385
Cumic Acid	1.839	9.472 × 10^−6^	3.197
valine	1.835	1.009 × 10^−5^	0.394
Cysteinylglycine	1.832	2.973 × 10^−5^	3.979
5,6-dihydrouracil	1.824	2.572 × 10^−5^	2.963
proline	1.812	2.147 × 10^−5^	0.359
adenosine	1.793	2.837 × 10^−5^	0.513
oxoproline	1.786	5.476 × 10^−5^	0.469
malonic acid	1.778	2.021 × 10^−4^	7.683
glycine	1.776	3.924 × 10^−5^	0.536
Maleamate	1.769	1.128 × 10^−4^	1.633
stearic acid	1.733	2.268 × 10^−4^	1.890
catechol	1.730	1.458 × 10^−4^	0.466
Adipamide	1.706	1.866 × 10^−4^	0.302
palmitic acid	1.704	4.068 × 10^−4^	1.719
maltotriose	1.703	2.545 × 10^−4^	0.573
Linoleic acid methyl ester	1.687	1.359 × 10^−3^	2.320
linolenic acid	1.682	9.211 × 10^−4^	1.930
MALONAMIDE	1.649	9.539 × 10^−4^	1.735
linoleic acid	1.639	2.797 × 10^−3^	1.646
creatine degr	1.620	1.159 × 10^−3^	1.702
Elaidic acid	1.563	4.451 × 10^−3^	1.568
sorbitol	1.559	2.184 × 10^−3^	1.583
glucose-6-phosphate	1.557	3.682 × 10^−3^	1.850
Methyl Palmitoleate	1.483	0.013	1,989
Monostearin	1.457	7.202 × 10^−3^	1.391
d-(glycerol 1-phosphate)	1.452	5.2941 × 10^−3^	0.687
caffeic acid	1.444	7.203 × 10^−3^	3.383
Phenylphosphoric acid	1.435	7.398 × 10^−3^	1.995
Maleimide	1.429	0.015	1.519
1-Monopalmitin	1.416	9.943 × 10^−3^	1.410
thymine	1.413	0.013	24.224
hexadecane	1.408	0.010	+
2-amino-2-methylpropane-1,3-diol	1.378	0.010	+
Nicotinoylglycine	1.370	0.011	+
Dioctyl phthalate	1.358	0.019	0.552
lysine	1.351	0.017	+
3-Hydroxyanthranilic acid	1.347	0.026	0.584
shikimic acid	1.330	0.016	1.446
arbutin	1.303	0.019	1.705
asparagine	1.291	0.019	+
maltitol	1.280	0.024	0.661
Glucose-1-phosphate	1.269	0.028	0.599
*N*-epsilon-Acetyl-l-lysine	1.234	0.038	0.682
Synephrine	1.206	0.034	0.674
3-Methyloxindole	1.199	0.045	2.544
Alizarin	1.198	0.041	0.370
Mono(2-ethylhexyl) phthalate	1.184	0.050	+

Note: The fold change = peak area of metabolites in ‘Dangshan Su’ fruit pollinated by ‘Wonhwang’ (DW)/peak area of metabolites in ‘Dangshan Su’ fruit pollinated by ‘Jingbaili’ (DJ); + indicates that the metabolite was only detected in DW.

**Table 2:** Differential metabolites at 63 DAPs in DW and DJ.

Metabolite	VIP	*p*-Value	Fold Change
Leucrose	1.918	5.181 × 10^−14^	-
Valine	1.907	2.145 × 10^−10^	5.355
Myo-inositol	1.766	4.069 × 10^−5^	0.682
Palatinitol	1.751	1.052 × 10^−4^	0.628
Sorbitol	1.720	1.402 × 10^−4^	2.293
Cellobiotol	1.702	2.840 × 10^−4^	0.622
Mannitol	1.651	7.616 × 10^−4^	0.478
Maltotriose	1.650	1.137 × 10^−3^	0.647
1-Methyladenosine	1.649	4.547 × 10^−4^	-
Lysine	1.646	5.772 × 10^−4^	-
Aspartic acid	1.643	5.372 × 10^−4^	0.557
2-Amino-2-methylpropane-1,3-diol	1.626	6.194 × 10^−4^	-
Elaidic acid	1.626	1.583 × 10^−3^	0.677
Glycine	1.625	1.313 × 10^−3^	1.396
Linoleic acid	1.558	4.088 × 10^−3^	0.629
Oxoproline	1.549	2.818 × 10^−3^	0.690
Galactinol	1.540	0.590 × 10^−3^	0.579
Sitosterol	1.510	6.632 × 10^−3^	0.702
*N*-epsilon-Acetyl-l-lysine	1.479	8.339 × 10^−3^	0.808
β-Mannosylglycerate	1.453	6.939 × 10^−3^	0.649
Ribitol	1.449	0.015	0.669
Adipamide	1.435	6.447 × 10^−3^	1.396
Fructose	1.423	0.010	0.615
Proline	1.411	0.017	0.472
Vanillylmandelic acid	1.386	0.019	0.715
Heptadecanoic acid	1.380	0.019	0.780
Gallic acid	1.379	0.024	0.605
Citramalic acid	1.346	0.016	+
3-Methyloxindole	1.288	0.025	0.727
d-Glyceric acid	1.272	0.045	1.384
3-Hydroxypropionic acid	1.211	0.043	0.566
Hydantoin, 5-(4-hydroxybutyl)-	1.194	0.049	-
Sorbose	1.196	0.050	-
2-Indanone	1.177	0.050	-

Note: The fold change = peak area of metabolites in DW/peak area of metabolites in DJ; + indicates that the metabolite was only detected in DW, and - indicates that the metabolite was only detected in DJ. Through Kyoto Encyclopedia of Genes and Genomes (KEGG) pathway analysis of differential metabolites between DW and DJ at 47 DAPs and 63 DAPs, 36 and 27 KEGG pathways were found to be enriched, respectively. Moreover, 31 KEGG pathways were significantly enriched (*p* < 0.05, FDR < 0.01) at 47 DAPs (Table 3), and they were mainly related to fatty acid metabolism, amino acid metabolism, and sugar metabolism. At 63 DAPs, the enrichment of the KEGG pathways was not significant.

**Table 3:** Significantly enriched KEGG pathways associated with differential metabolites.

KEGG Pathway	*p*-Value	FDR
Glycerophospholipid metabolism	5.330 × 10^−8^	9.592 × 10^−7^
Glycerolipid metabolism	5.330 × 10^−8^	9.592 × 10^−7^
α-Linolenic acid metabolism	2.098 × 10^−7^	2.518 × 10^−6^
Glutathione metabolism	6.650 × 10^−7^	2.946 × 10^−6^
Biosynthesis of unsaturated fatty acids	8.173 × 10^−7^	2.946 × 10^−6^
Pyrimidine metabolism	8.620 × 10^−7^	2.946 × 10^−6^
Pantothenate and CoA biosynthesis	8.805 × 10^−7^	2.946 × 10^−6^
Fatty acid biosynthesis	1.010 × 10^−6^	2.946 × 10^−6^
Fatty acid elongation in mitochondria	1.080 × 10^−6^	2.946 × 10^−6^
Fatty acid metabolism	1.080 × 10^−6^	2.946 × 10^−6^
β-Alanine metabolism	1.124 × 10^−6^	2.946 × 10^−6^
Phenylalanine, tyrosine and tryptophan biosynthesis	1.130 × 10^−6^	2.946 × 10^−6^
Fructose and mannose metabolism	1.191 × 10^−6^	2.946 × 10^−6^
Valine, leucine and isoleucine biosynthesis	1.309 × 10^−6^	2.946 × 10^−6^
Valine, leucine and isoleucine degradation	1.309 × 10^−6^	2.946 × 10^−6^
Glucosinolate biosynthesis	1.309 × 10^−6^	2.946 × 10^−6^
Arginine and proline metabolism	1.802 × 10^−6^	3.280 × 10^−6^
Cysteine and methionine metabolism	1.823 × 10^−6^	3.280 × 10^−6^
Carbon fixation in photosynthetic organisms	1.823 × 10^−6^	3.280 × 10^−6^
Nicotinate and nicotinamide metabolism	1.823 × 10^−6^	3.280 × 10^−6^
Cyanoamino acid metabolism	1.968 × 10^−6^	3.374 × 10^−6^
Galactose metabolism	2.552 × 10^−6^	4.18 × 10^−6^
Tryptophan metabolism	4.674 × 10^−6^	7.32 × 10^−6^
Methane metabolism	7.196 × 10^−6^	1.04 × 10^−5^
Nitrogen metabolism	7.196 × 10^−6^	1.04 × 10^−5^
Glycine, serine and threonine metabolism	2.417 × 10^−5^	3.35 × 10^−5^
Starch and sucrose metabolism	1.051 × 10^−4^	1.261 × 10^−4^
Amino sugar and nucleotide sugar metabolism	1.051 × 10^−4^	1.261 × 10^−4^
Glycolysis or Gluconeogenesis	1.051 × 10^−4^	1.261 × 10^−4^
Pentose and glucuronate interconversions	1.051 × 10^−4^	1.261 × 10^−4^
Lysine biosynthesis	6.498 × 10^−3^	7.546 × 10^−3^

**Table 4:** The differential intermediate metabolites in lignin synthesis.

Metabolite	47 DAPs		63 DAPs	
	VIP	*p*-Value	VIP	*p*-Value
*p*-coumaric acid	1.08325	0.00018 **	1.90639	0.01701 *
Caffeic acid	0.776344	0.11483	0.483629	0.66653
Ferulic acid	1.063	0.00129 **	0.182974	0.87196
Coniferaldehyde	0.839268	0.07556	0.766778	0.48518
Sinapaldehyde	1.07733	0.00040 **	0.444275	0.69285
Coniferyl alcohol	1.04866	0.00268 **	1.50585	0.11830
Sinapyl alcohol	1.06264	0.00132 **	0.213007	0.85107

Paired *t*-test (Paired *t*-test); ** indicates significant difference at the 0.01 level; * indicates significant difference at the 0.05 level.

**Table 5:** MRM detection parameters for intermediate metabolites of lignin synthesis.

Metabolite	Ion Mode	Precursor Ion (*m*/*z*)	Product Ion (*m*/*z*)	DP	CE
Cinnamic acid	-	146.9	103.0	−30	−13
*p*-coumaric acid	-	162.8	119.0	−30	−15.5
Caffeic acid	-	179.0	135.0	−30	−20
Ferulic acid	-	193.0	134.0	−30	−20
			149.1	−30	−13
			116.9	−30	−20
Sinapic acid	-	223.0	148.9	−30	−17
			164.0	−30	−18
Coniferaldehyde	-	177.0	162.0	−30	−17
			134.0	−30	−28
Sinapaldehyde	-	207.0	192.0	−24	−17
			176.8	−30	−27
			149.2	−30	−35
Coniferyl alcohol	-	179.1	146.0	−30	−19
			164.0	−30	−18
			161	−30	−13
Sinapyl alcohol	-	209.0	194.0	−30	−17
			179.0	−40	−24
			176.1	−40	−18

**Table 6:** Standard information and standard curves for intermediate metabolites of lignin synthesis.

Metabolite	Chemical Formula	Formula Weight	Standard Curve	Correlation Coefficient	Retention Time (min)
Cinnamic acid	C_9_H_8_O_2_	148.161	Y = 1.12 × 10^3^X + 4.35 × 10^4^	R^2^ = 1.0000	11.8
*p*-coumaric acid	C_9_H_8_O_3_	164.160	Y = 2.36 × 10^4^X + 2.29 × 10^5^	R^2^ = 0.9994	10.4
Caffeic acid	C_9_H_8_O_4_	180.159	Y = 4.57 × 10^4^X − 1.33 × 10^5^	R^2^ = 0.9995	9.9
Ferulic acid	C_10_H_10_O_4_	194.186	Y = 8.38 × 10^3^X + 6.2 × 10^3^	R^2^ = 0.9999	10.5
Sinapic acid	C_11_H_12_O_5_	224.212	Y = 455X − 603	R^2^ = 0.9992	10.3
Coniferaldehyde	C_10_H_10_O_3_	178.187	Y = 2.38 × 10^4^X + 6.08 × 10^4^	R^2^ = 0.9998	11.0
Sinapaldehyde	C_11_H_12_O_4_	208.213	Y = 1.22 × 10^4^X − 2.55 × 10^4^	R^2^ = 0.9999	10.9
Coniferyl alcohol	C_10_H_12_O_3_	180.203	Y = 98.4X − 519	R^2^ = 0.9998	10.3
Sinapyl alcohol	C_11_H_14_O_4_	210.229	Y = 150X − 776	R^2^ = 0.9998	10.1

## Supplementary Materials

Supplementary materials can be found at http://www.mdpi.com/1422-0067/19/8/2273/s1.
