# Supplementary material for: Effects of Different Pollens on Primary Metabolism and Lignin Biosynthesis in Pear
Source: Int J Mol Sci. 2018 Aug 2;19(8):2273. doi: 10.3390/ijms19082273 (PMC6122022; doi:10.3390/ijms19082273)
Supplement: Supplementary file 1 [file ijms-19-02273-s001.pdf]

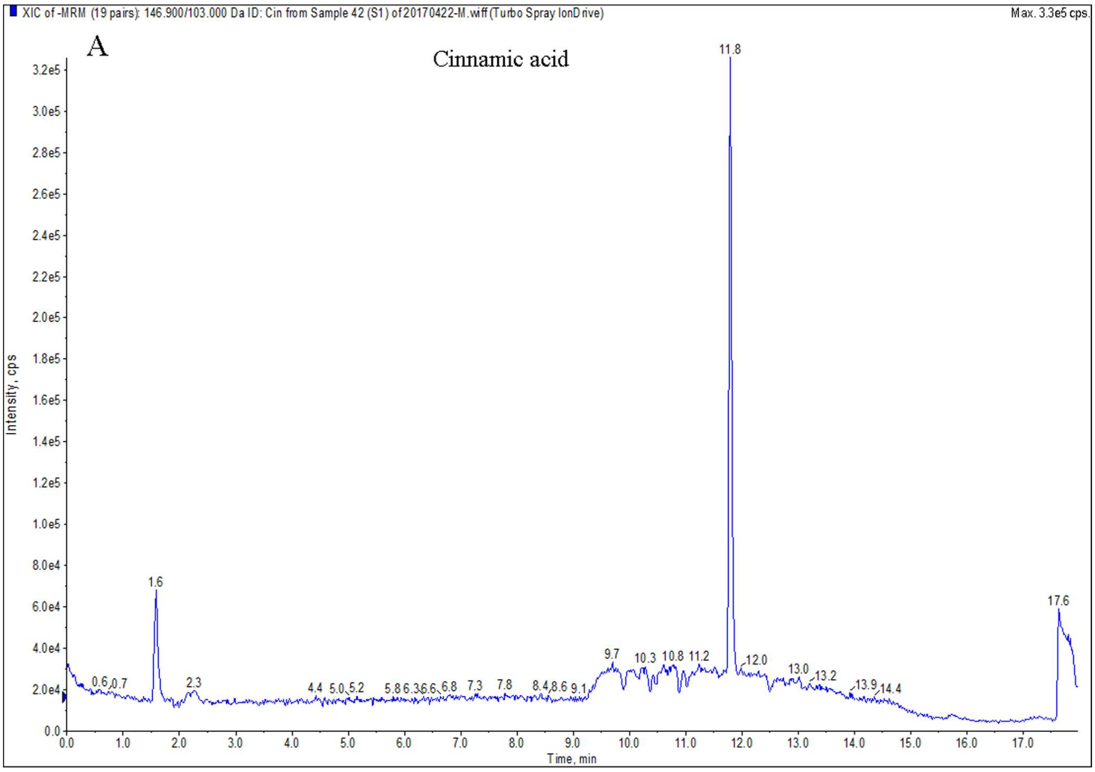
XIC of -MRM (19 pairs): 146.900/103.000 Da ID: Cin from Sample 42 (S1) of 20170422-M.wiff (Turbo Spray IonDrive)
Max. 3.3e5 cps.
A
Cinnamic acid
Intensity, cps
Time, min

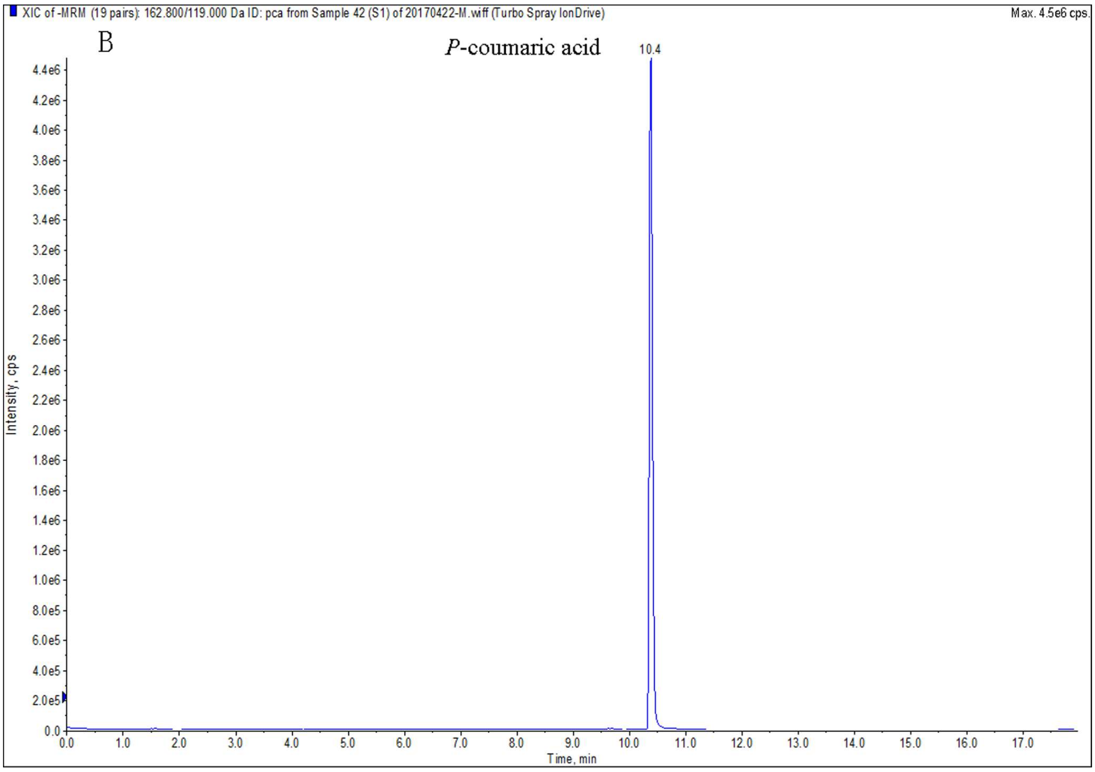
XIC of -MRM (19 pairs): 162.800/119.000 Da ID: pca from Sample 42 (S1) of 20170422-M.wiff (Turbo Spray IonDrive)
Max. 4.5e6 cps.
B
P-coumaric acid
Intensity, cps
Time, min

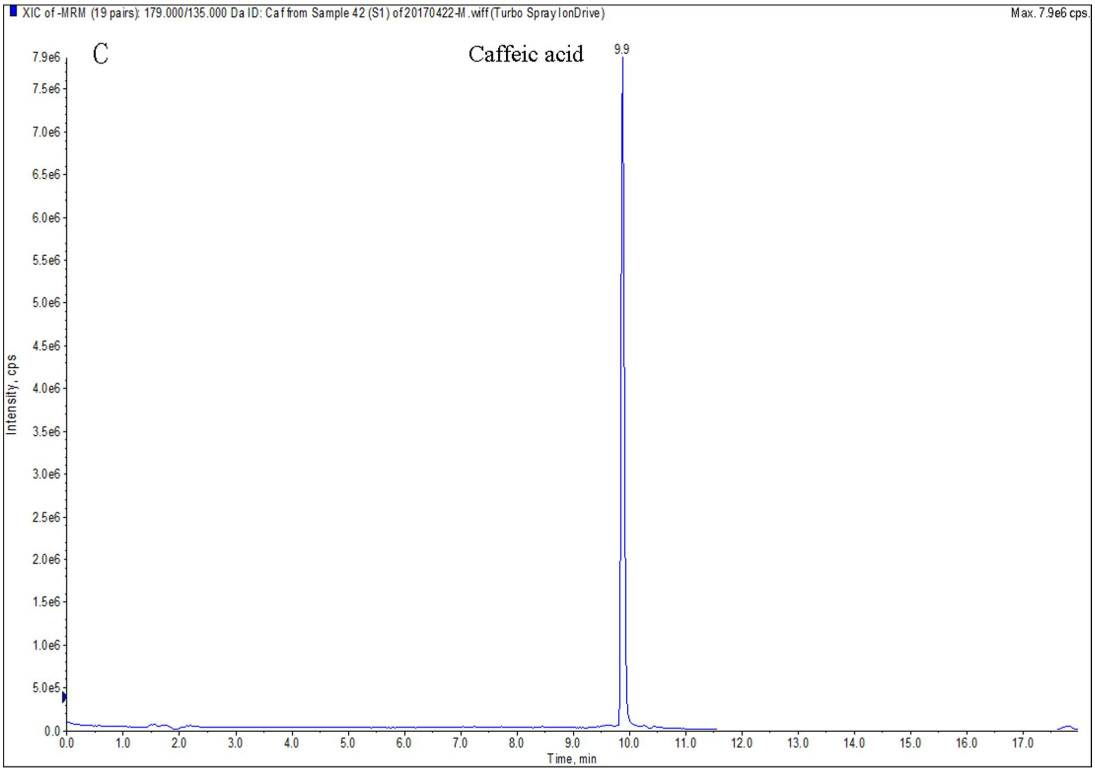
XIC of -MRM (19 pairs): 179.000/135.000 Da ID: Caf from Sample 42 (S1) of 20170422-M.wiff (Turbo Spray IonDrive)
Max. 7.9e6 cps.
C
Caffeic acid
9.9
Intensity, cps
Time, min

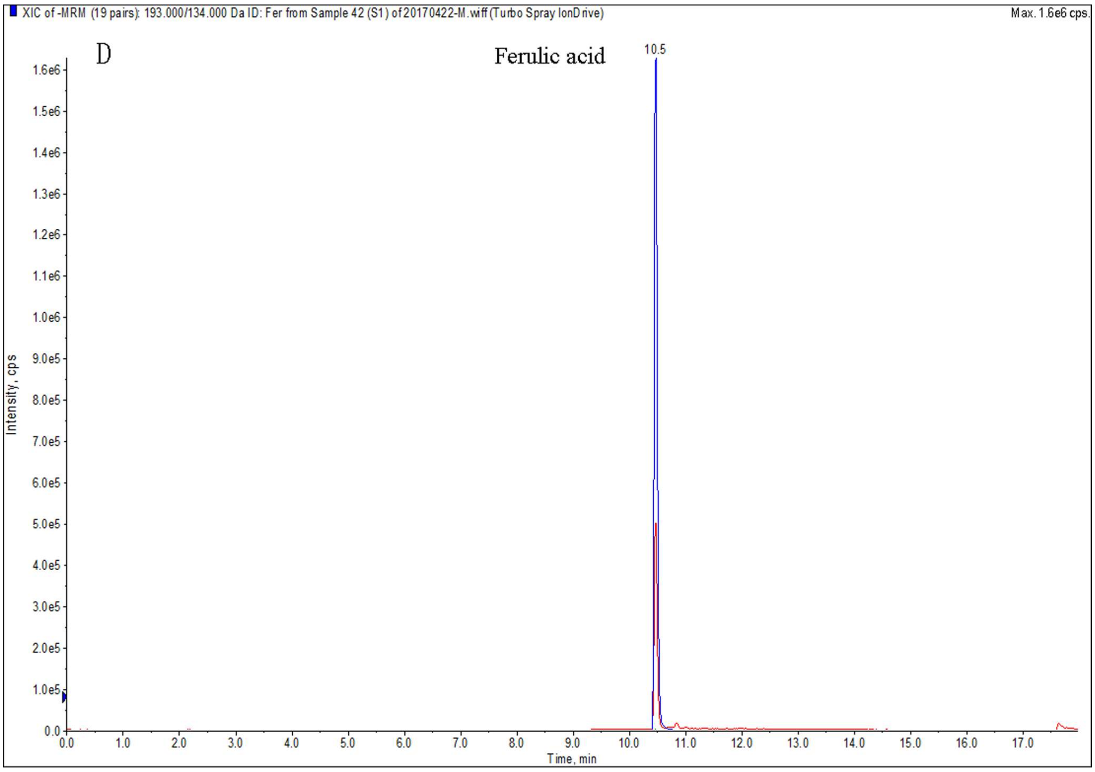
XIC of -MRM (19 pairs): 193.000/134.000 Da ID: Fer from Sample 42 (S1) of 20170422-M.wiff (Turbo Spray IonDrive)
Max. 1.6e6 cps.
D
Ferulic acid
10.5
Intensity, cps
Time, min

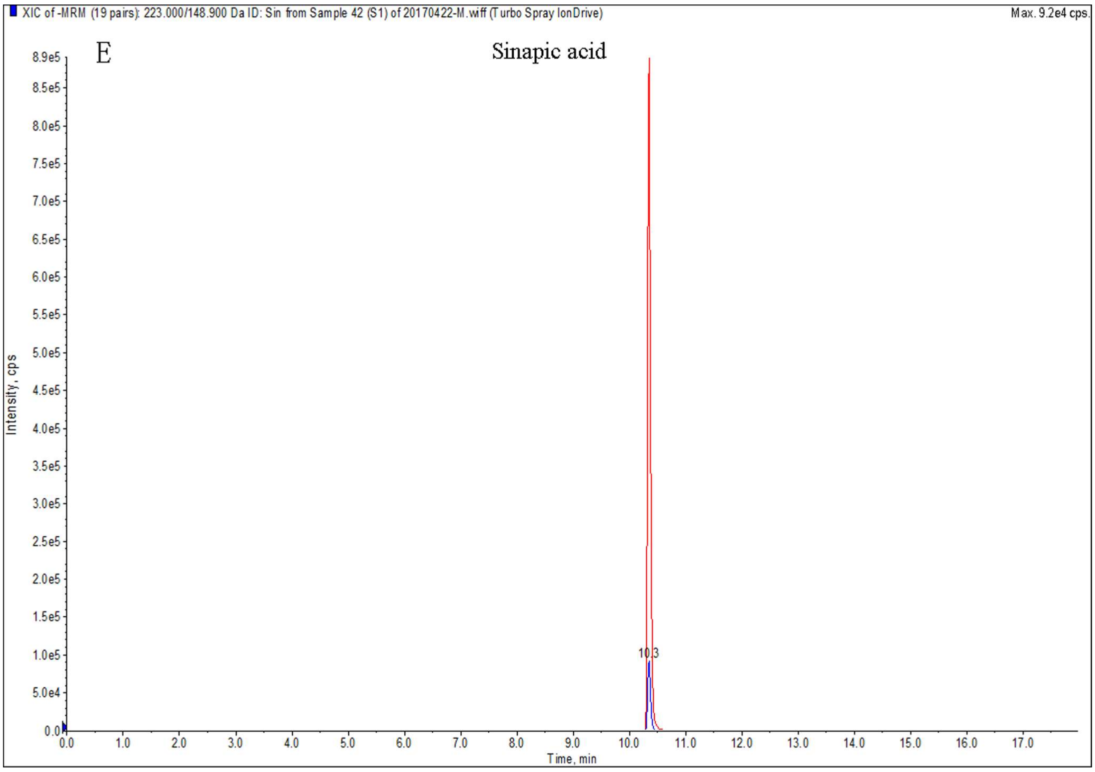
XIC of -MRM (19 pairs): 223.000/148.900 Da ID: Sin from Sample 42 (S1) of 20170422-M.wiff (Turbo Spray IonDrive)
Max. 9.2e4 cps.
E
Sinapic acid
10.3
Intensity, cps
Time, min

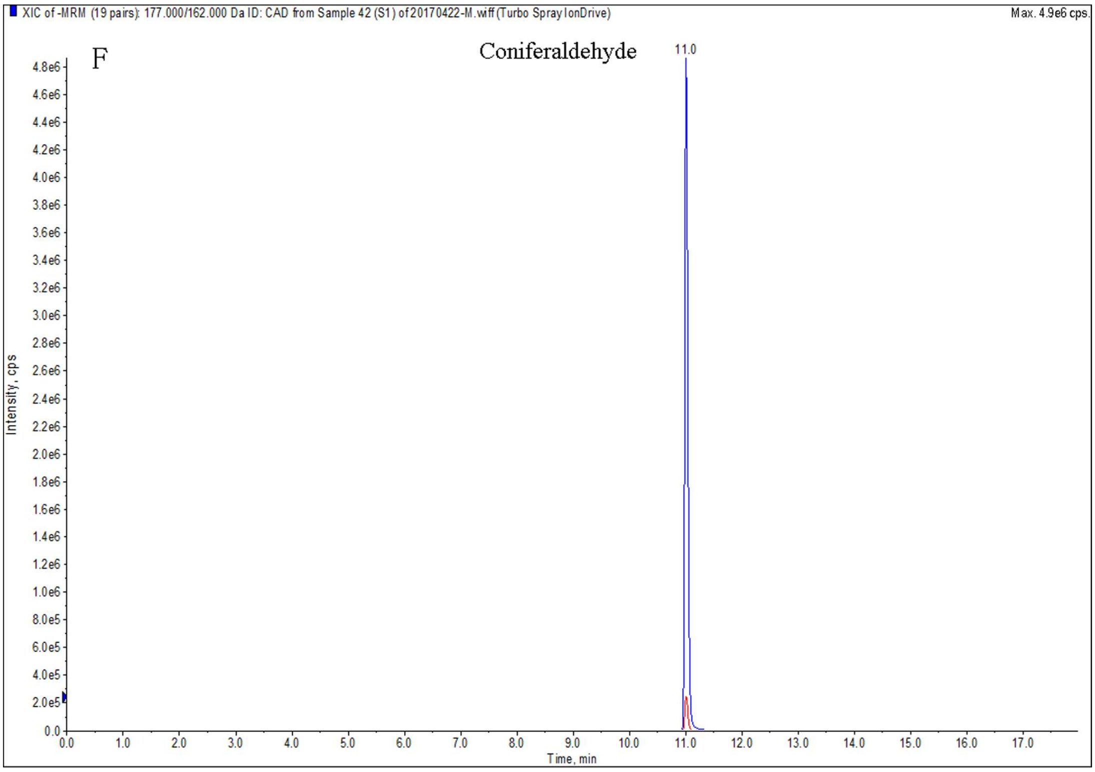
XIC of -MRM (19 pairs): 177.000/162.000 Da ID: CAD from Sample 42 (S1) of 20170422-M.wiff (Turbo Spray IonDrive)
Max. 4.9e6 cps.
F
Coniferaldehyde
11.0
Intensity, cps
Time, min

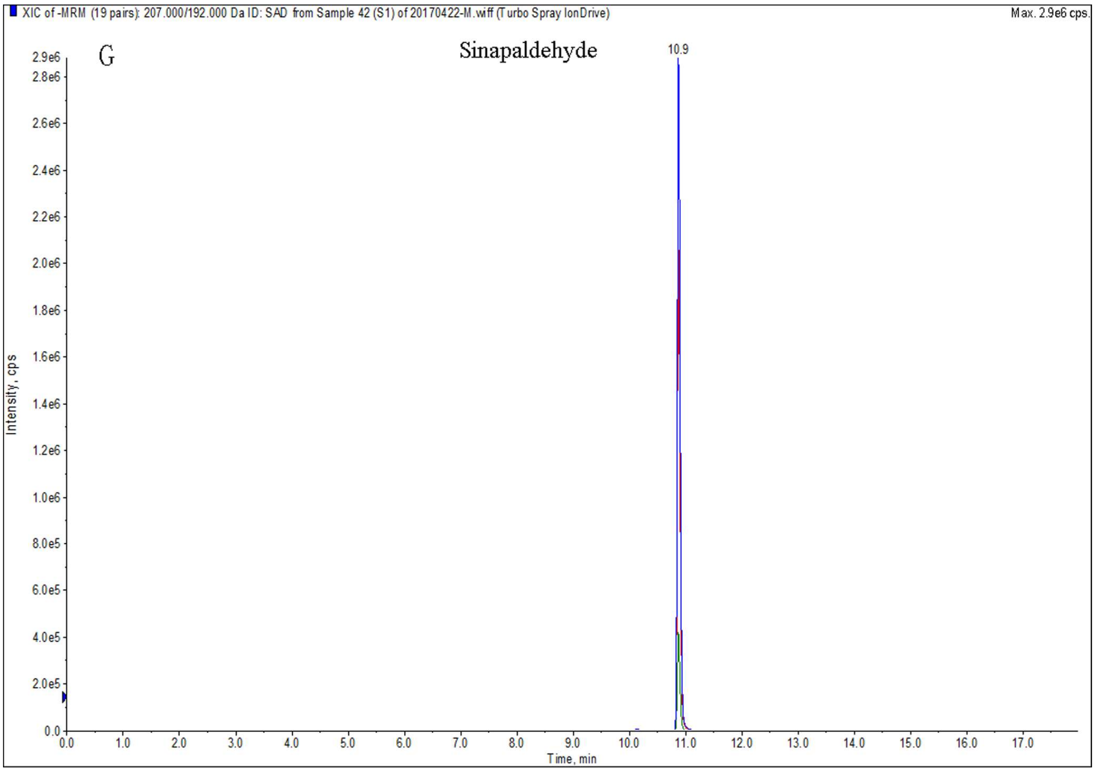
XIC of -MRM (19 pairs): 207.000/192.000 Da ID: SAD from Sample 42 (S1) of 20170422-M.wiff (Turbo Spray IonDrive)
Max. 2.9e6 cps.
G
Sinapaldehyde
10.9
Intensity, cps
Time, min

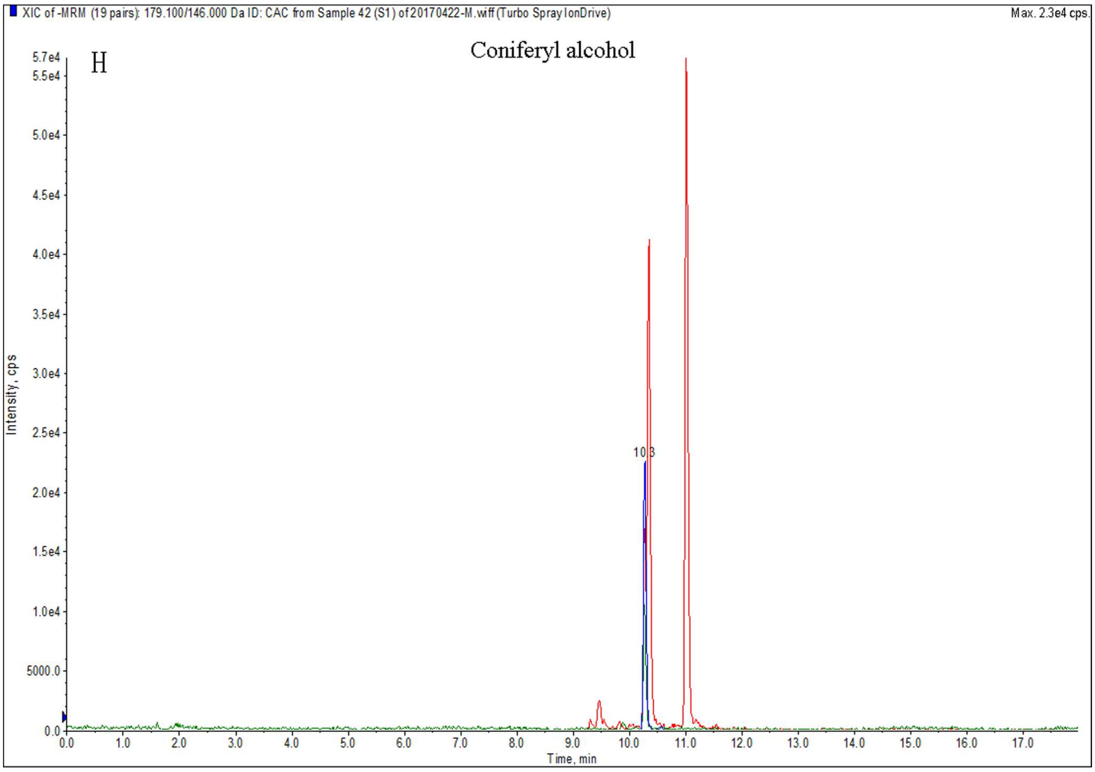
XIC of -MRM (19 pairs): 179.100/146.000 Da ID: CAC from Sample 42 (S1) of 20170422-M.wiff (Turbo Spray IonDrive)
Max. 2.3e4 cps.
H
Coniferyl alcohol
Intensity, cps
Time, min

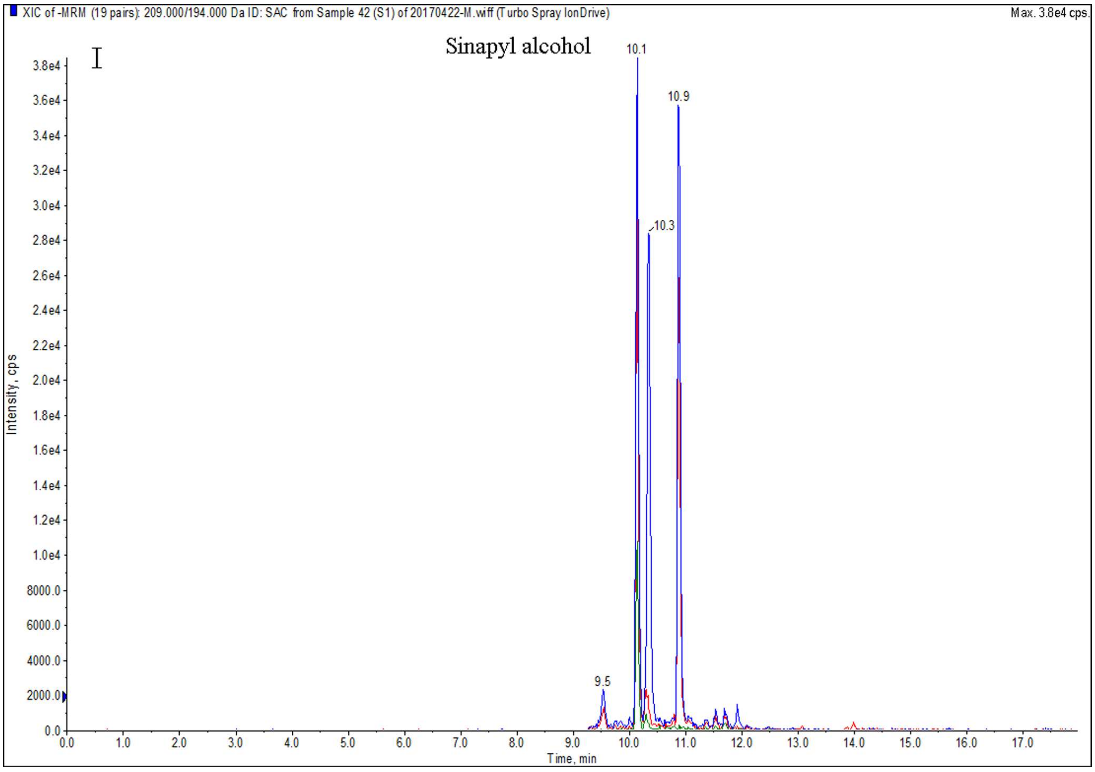
XIC of -MRM (19 pairs): 209.000/194.000 Da ID: SAC from Sample 42 (S1) of 20170422-M.wiff (Turbo Spray IonDrive)
Max. 3.8e4 cps.
I
Sinapyl alcohol
Intensity, cps
Time, min

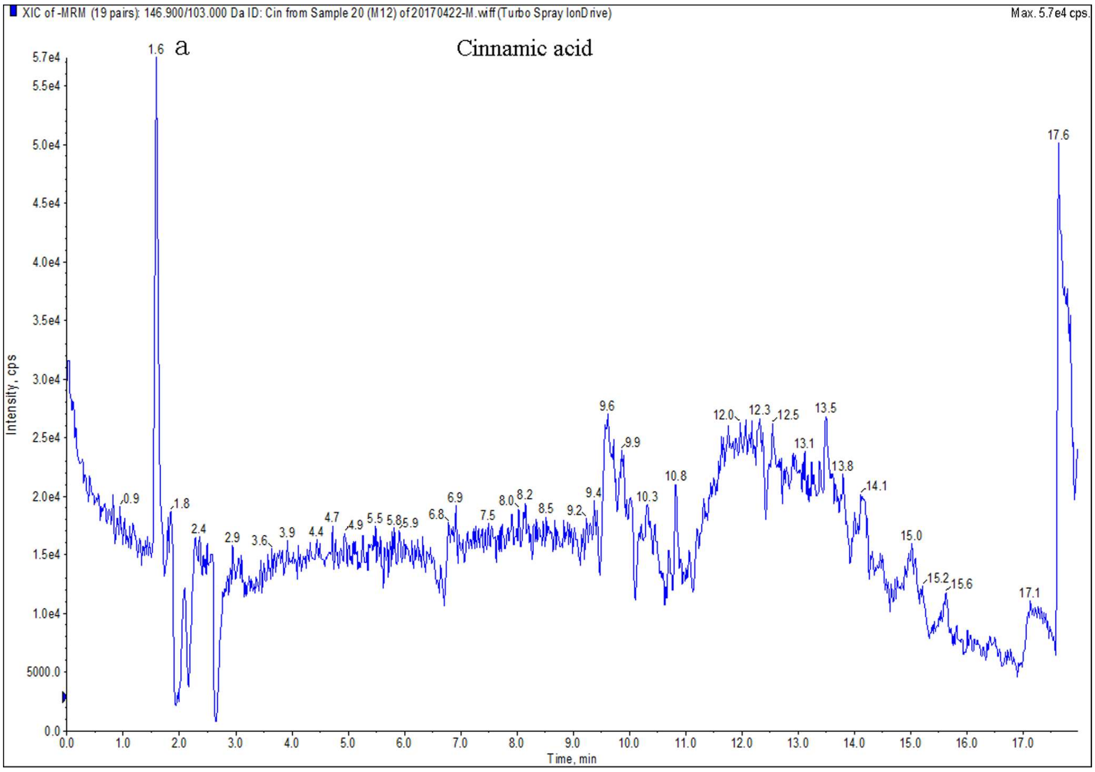
XIC of -MRM (19 pairs): 146.900/103.000 Da ID: Cin from Sample 20 (M12) of 20170422-M.wiff (Turbo Spray IonDrive)
Max. 5.7e4 cps.
a
Cinnamic acid
Intensity, cps
Time, min

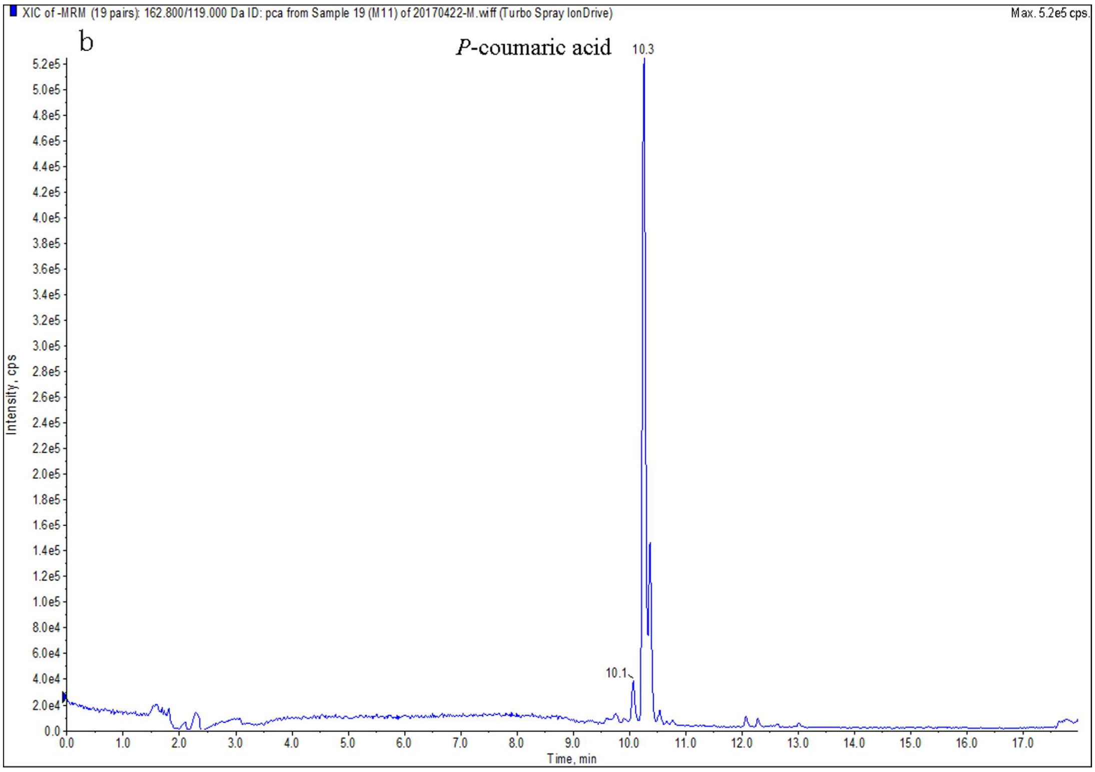
XIC of -MRM (19 pairs): 162.800/119.000 Da ID: pca from Sample 19 (M11) of 20170422-M.wiff (Turbo Spray IonDrive)
Max. 5.2e5 cps.
b
P-coumaric acid
10.3
10.1
Intensity, cps
Time, min

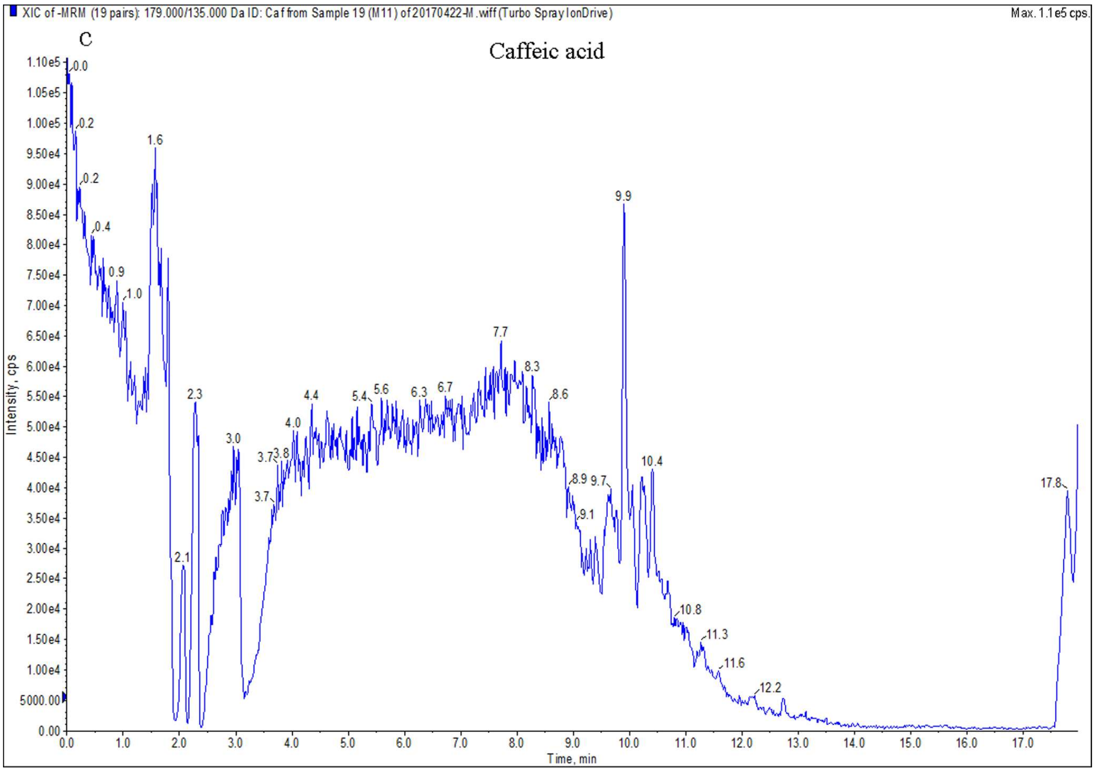
XIC of -MRM (19 pairs): 179.000/135.000 Da ID: Caf from Sample 19 (M11) of 20170422-M.wiff (Turbo Spray IonDrive)
Max. 1.1e5 cps.
c
Caffeic acid
Intensity, cps
Time, min

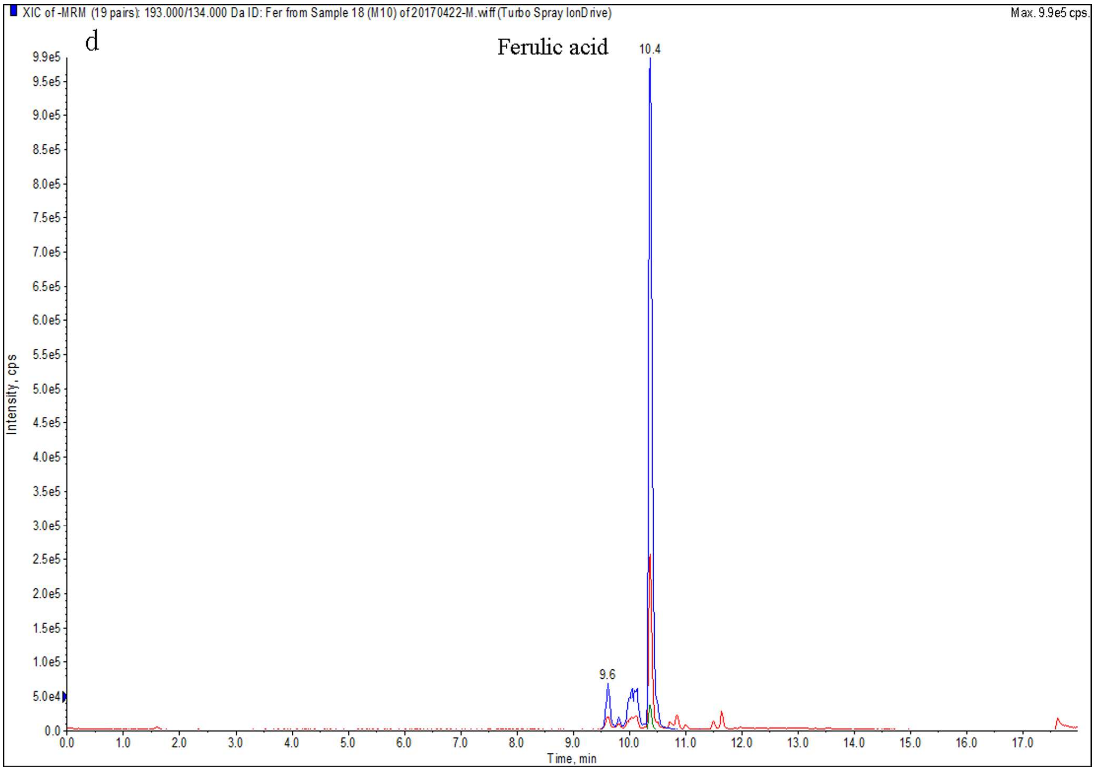
XIC of -MRM (19 pairs): 193.000/134.000 Da ID: Fer from Sample 18 (M10) of 20170422-M.wiff (Turbo Spray IonDrive)
Max. 9.9e5 cps.
d
Ferulic acid
Intensity, cps
Time, min

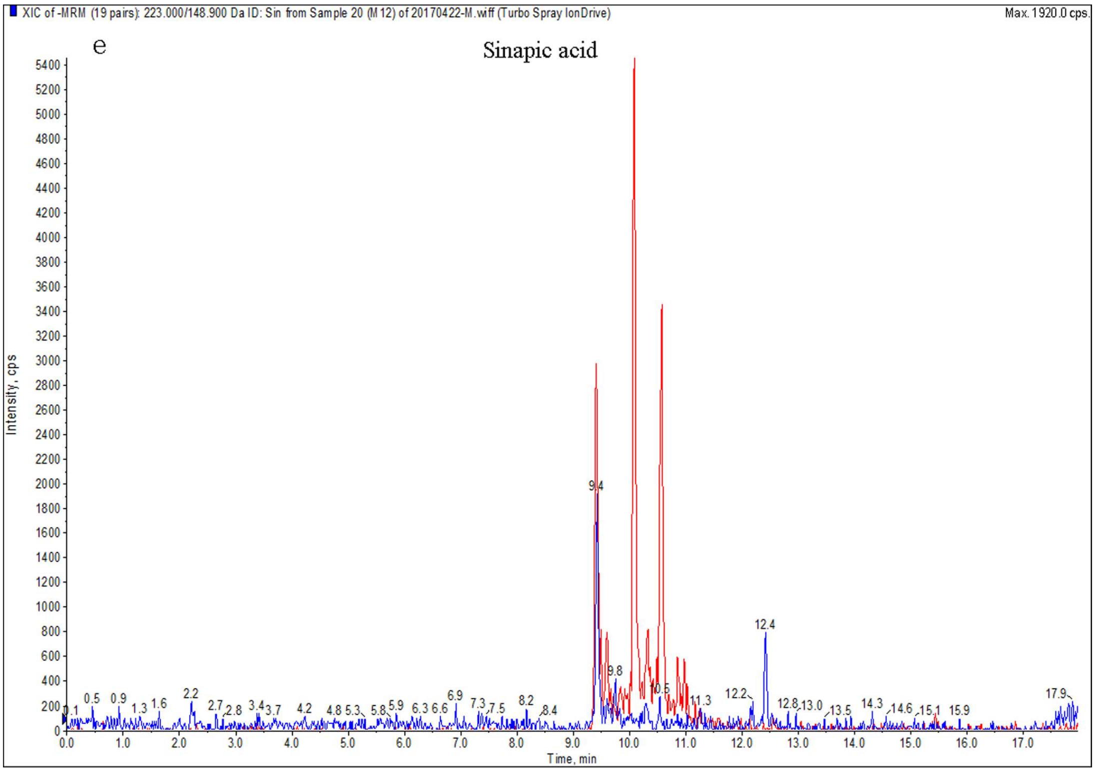
XIC of -MRM (19 pairs): 223.000/148.900 Da ID: Sin from Sample 20 (M12) of 20170422-M.wiff (Turbo Spray IonDrive)
Max. 1920.0 cps.
e
Sinapic acid
Intensity, cps
Time, min

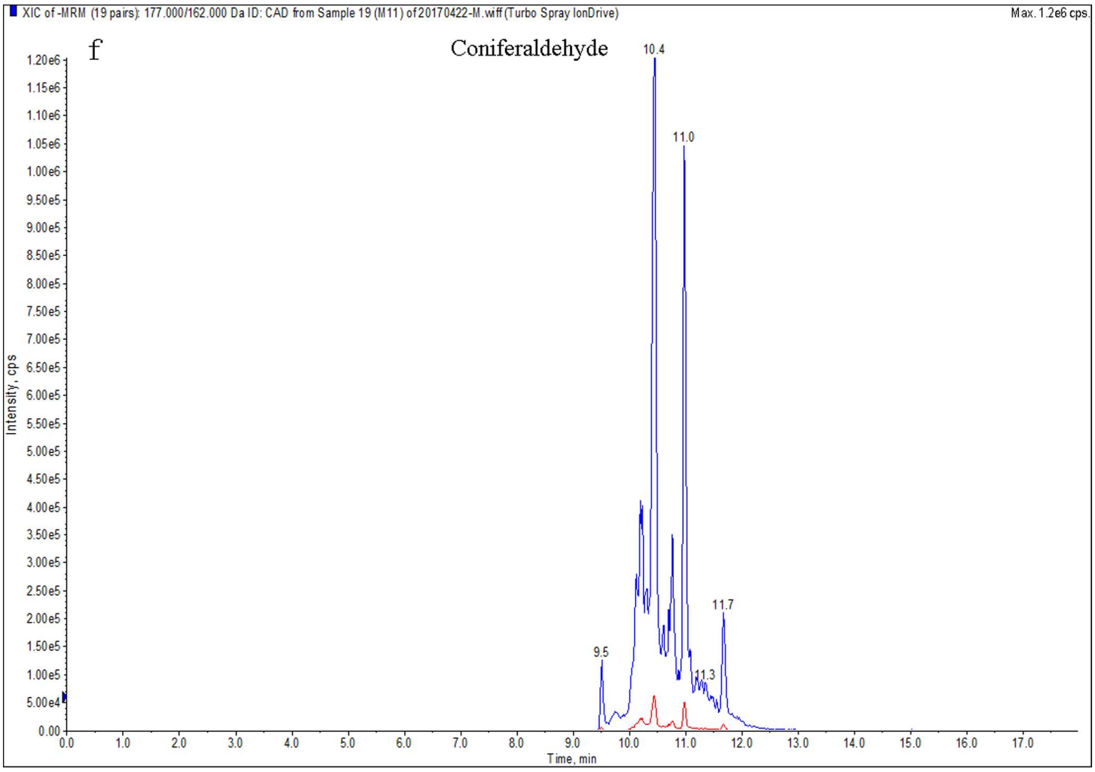

XIC of -MRM (19 pairs): 177.000/162.000 Da ID: CAD from Sample 19 (M11) of 20170422-M.wiff (Turbo Spray IonDrive)
Max. 1.2e6 cps.
f
Coniferaldehyde
Intensity, cps
Time, min
10.4
11.0
11.7
9.5
11.3

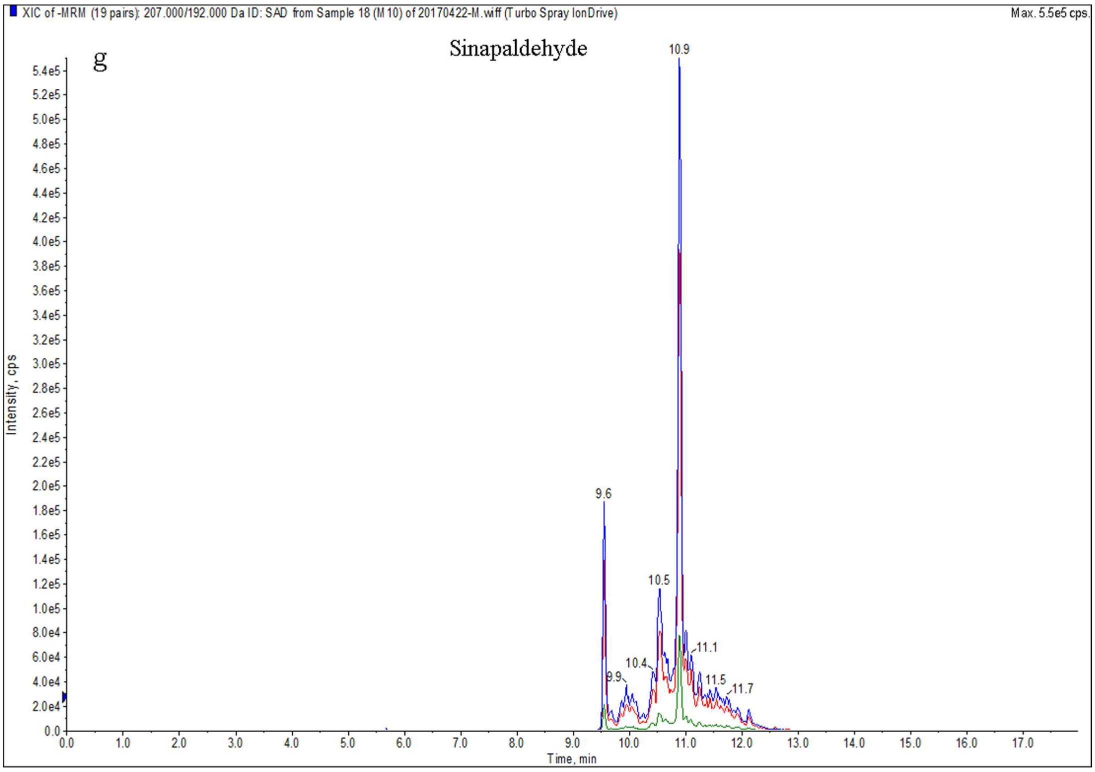

XIC of -MRM (19 pairs): 207.000/192.000 Da ID: SAD from Sample 18 (M10) of 20170422-M.wiff (Turbo Spray IonDrive)
Max. 5.5e5 cps.
g
Sinapaldehyde
Intensity, cps
Time, min
10.9
9.6
10.5
10.4
9.9
11.1
11.5
11.7

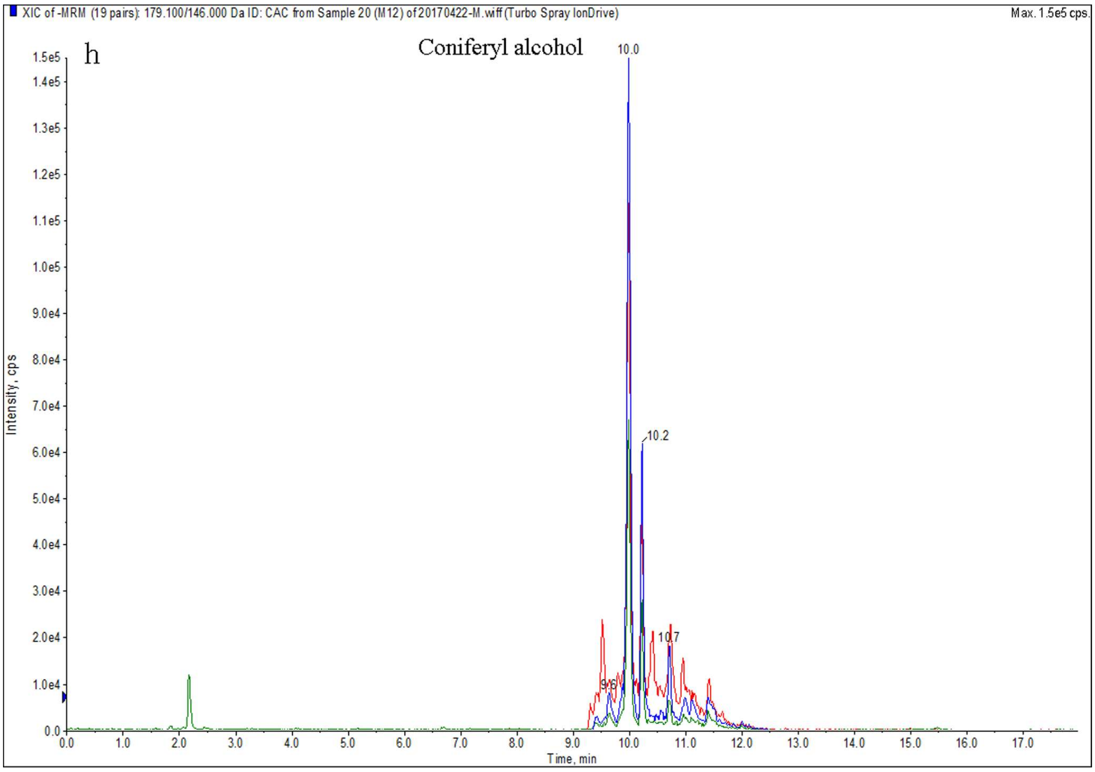

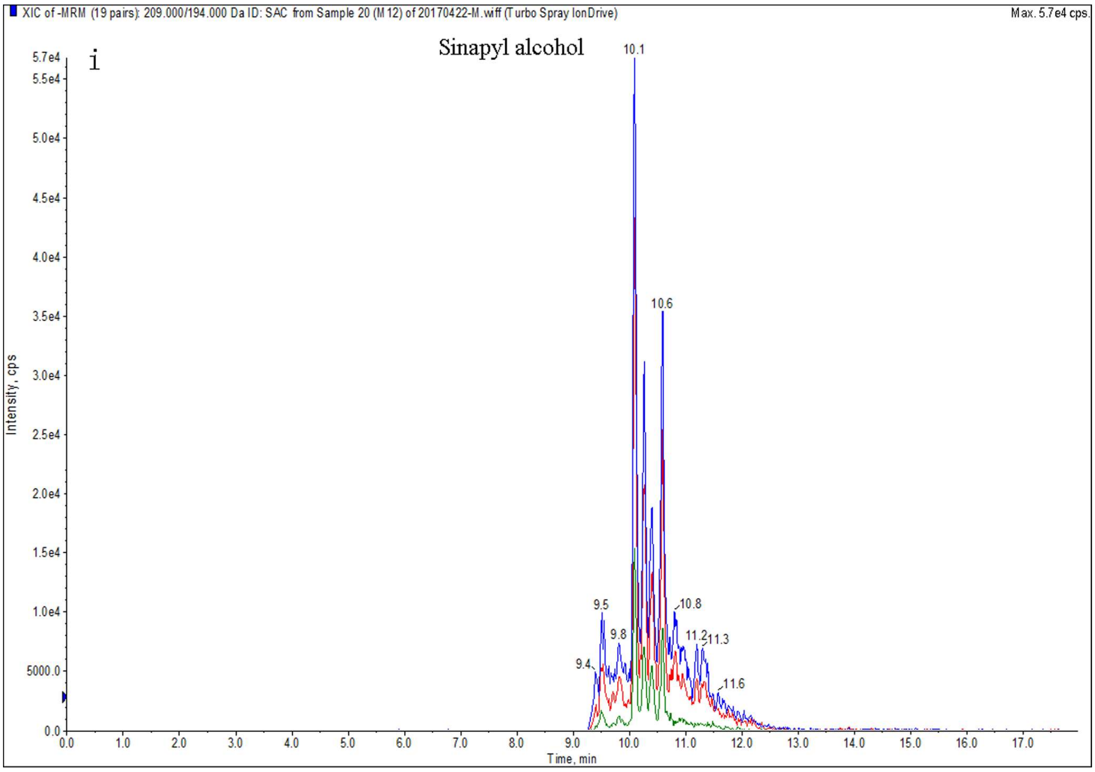

Figure S1 Ion chromatograms of lignin synthesis intermediate metabolites

Note A-I: standards; a-i: samples;

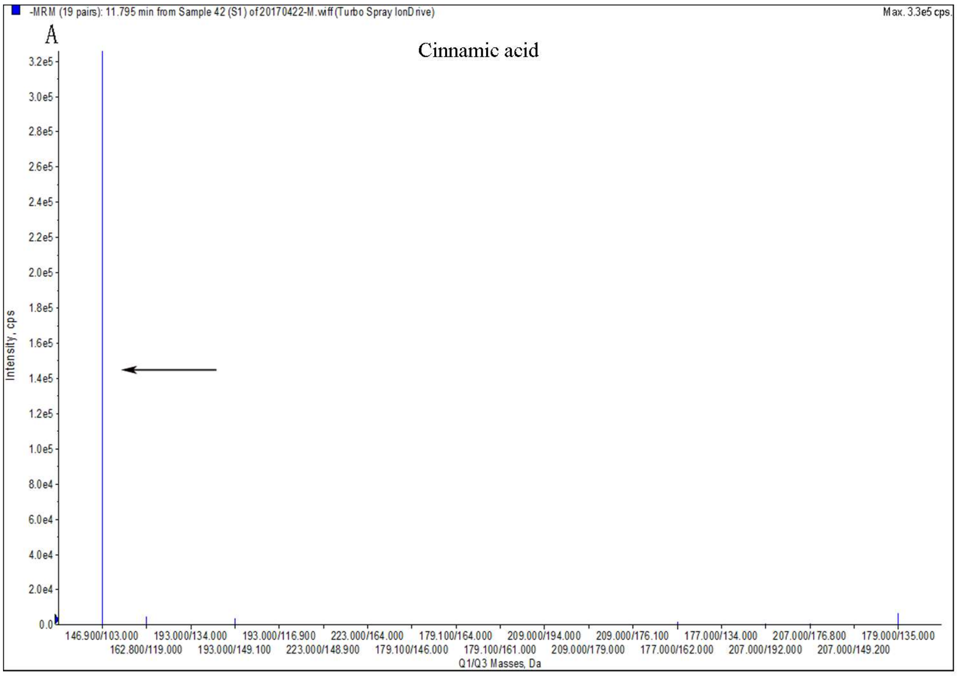
-MRM (19 pairs): 11.795 min from Sample 42 (S1) of 20170422-M.wiff (Turbo Spray IonDrive)
Max. 3.3e5 cps.
A
Cinnamic acid
Intensity, cps
146.900/103.000
162.800/119.000
193.000/134.000
193.000/149.100
193.000/116.900
223.000/148.900
223.000/164.000
179.100/146.000
179.100/164.000
179.100/161.000
209.000/194.000
209.000/179.000
209.000/176.100
177.000/162.000
177.000/134.000
207.000/192.000
207.000/176.800
207.000/149.200
179.000/135.000
Q1/Q3 Masses, Da

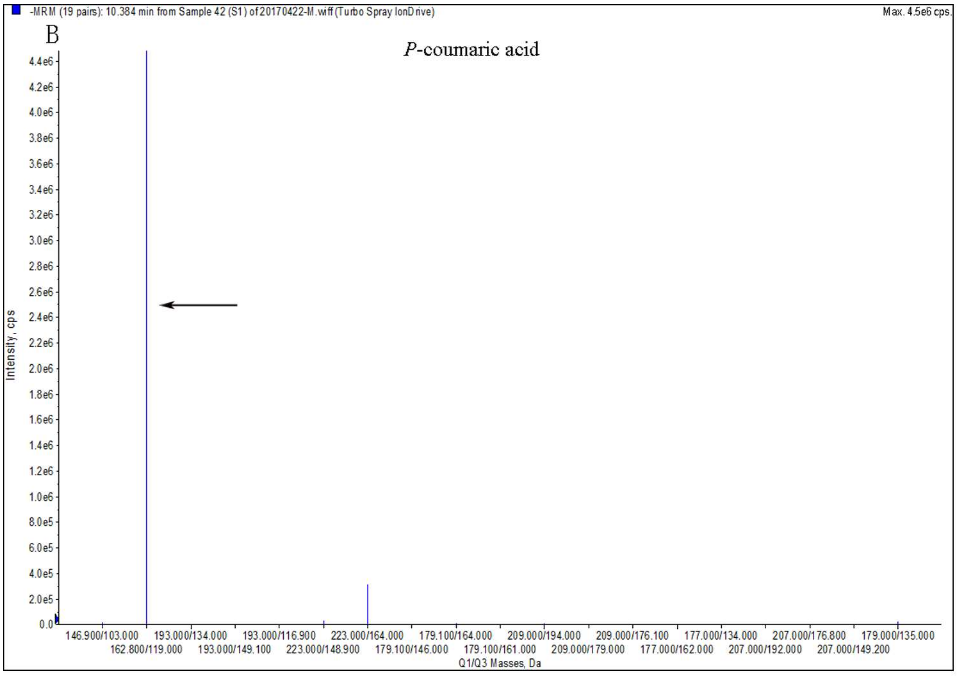
-MRM (19 pairs): 10.384 min from Sample 42 (S1) of 20170422-M.wiff (Turbo Spray IonDrive)
Max. 4.5e6 cps.
B
P-coumaric acid
Intensity, cps
146.900/103.000
162.800/119.000
193.000/134.000
193.000/149.100
193.000/116.900
223.000/148.900
223.000/164.000
179.100/146.000
179.100/164.000
179.100/161.000
209.000/194.000
209.000/179.000
209.000/176.100
177.000/162.000
177.000/134.000
207.000/192.000
207.000/176.800
207.000/149.200
179.000/135.000
Q1/Q3 Masses, Da

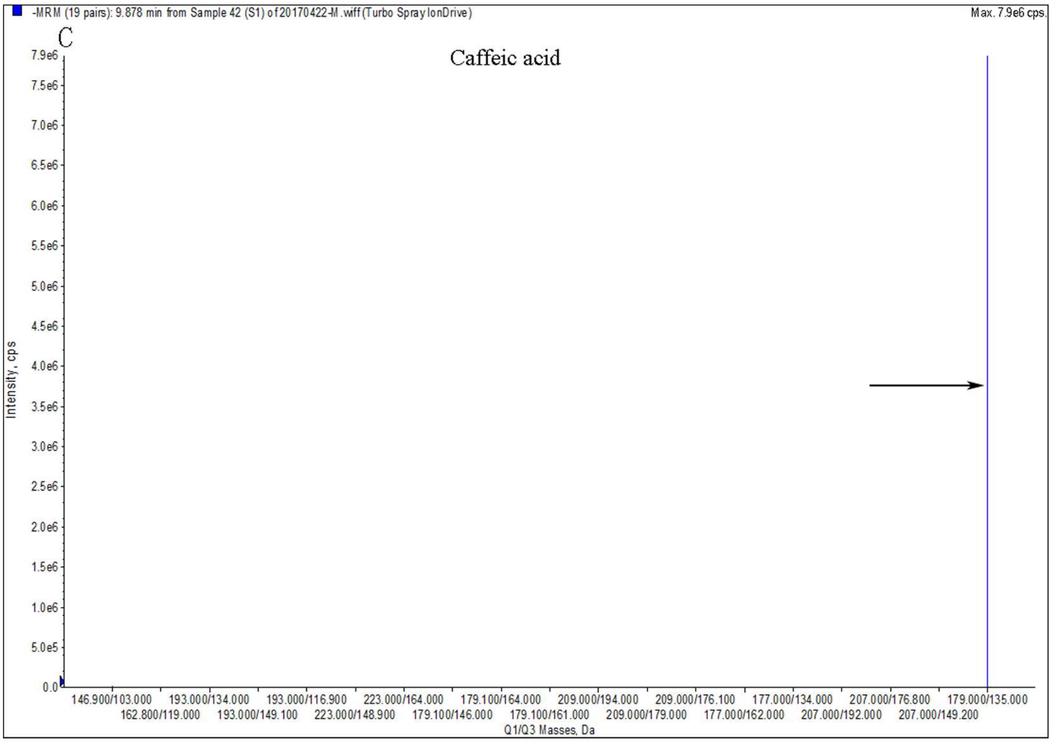
-MRM (19 pairs): 9.878 min from Sample 42 (S1) of 20170422-M.wiff (Turbo Spray IonDrive)
Max. 7.9e6 cps.
C
Caffeic acid
Intensity, cps
Q1/Q3 Masses, Da

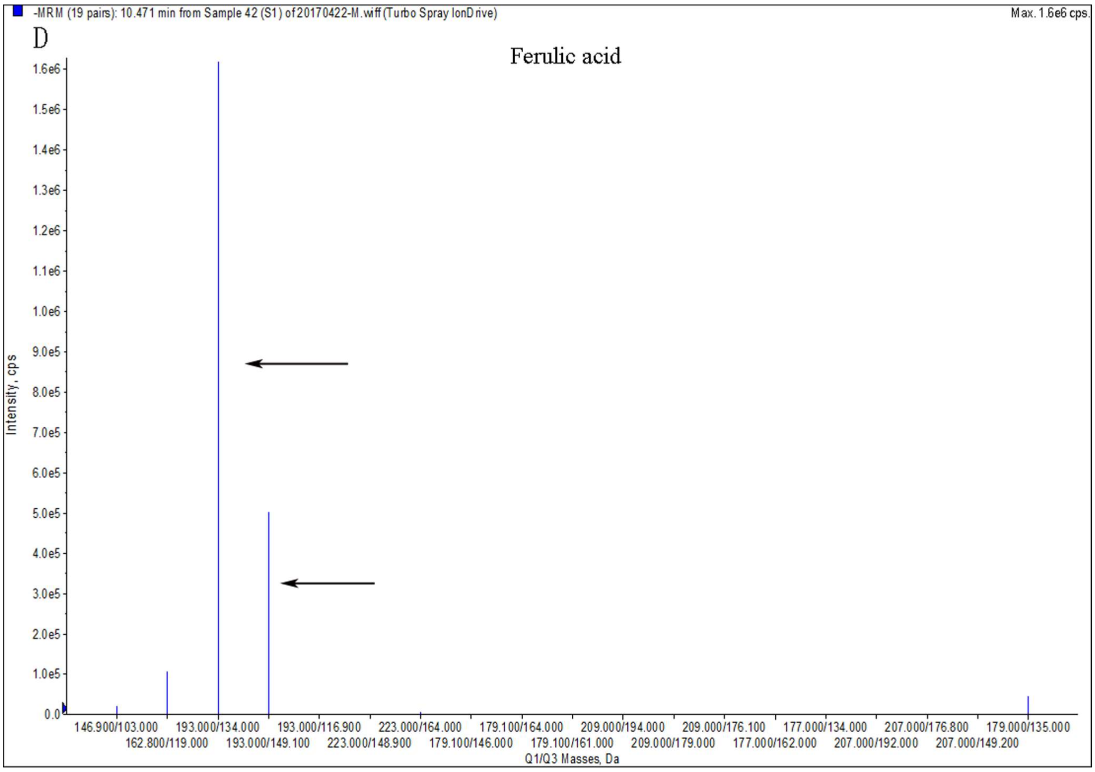
-MRM (19 pairs): 10.471 min from Sample 42 (S1) of 20170422-M.wiff (Turbo Spray IonDrive)
Max. 1.6e6 cps.
D
Ferulic acid
Intensity, cps
Q1/Q3 Masses, Da

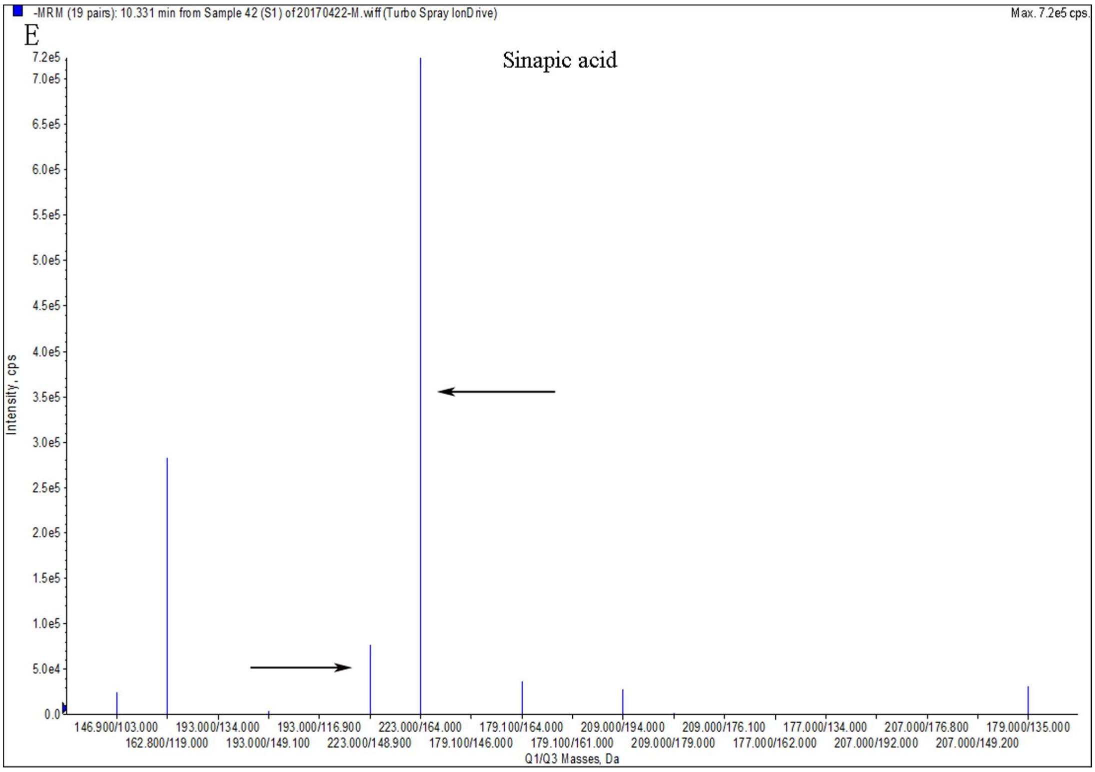
-MRM (19 pairs): 10.331 min from Sample 42 (S1) of 20170422-M.wiff (Turbo Spray IonDrive)
Max. 7.2e5 cps.
E
Sinapic acid
Intensity, cps
7.2e5
7.0e5
6.5e5
6.0e5
5.5e5
5.0e5
4.5e5
4.0e5
3.5e5
3.0e5
2.5e5
2.0e5
1.5e5
1.0e5
5.0e4
0.0
146.900/103.000
162.800/119.000
193.000/134.000
193.000/149.100
193.000/116.900
223.000/148.900
223.000/164.000
179.100/146.000
179.100/164.000
179.100/161.000
209.000/194.000
209.000/179.000
209.000/176.100
177.000/162.000
177.000/134.000
207.000/192.000
207.000/176.800
207.000/149.200
179.000/135.000
Q1/Q3 Masses, Da

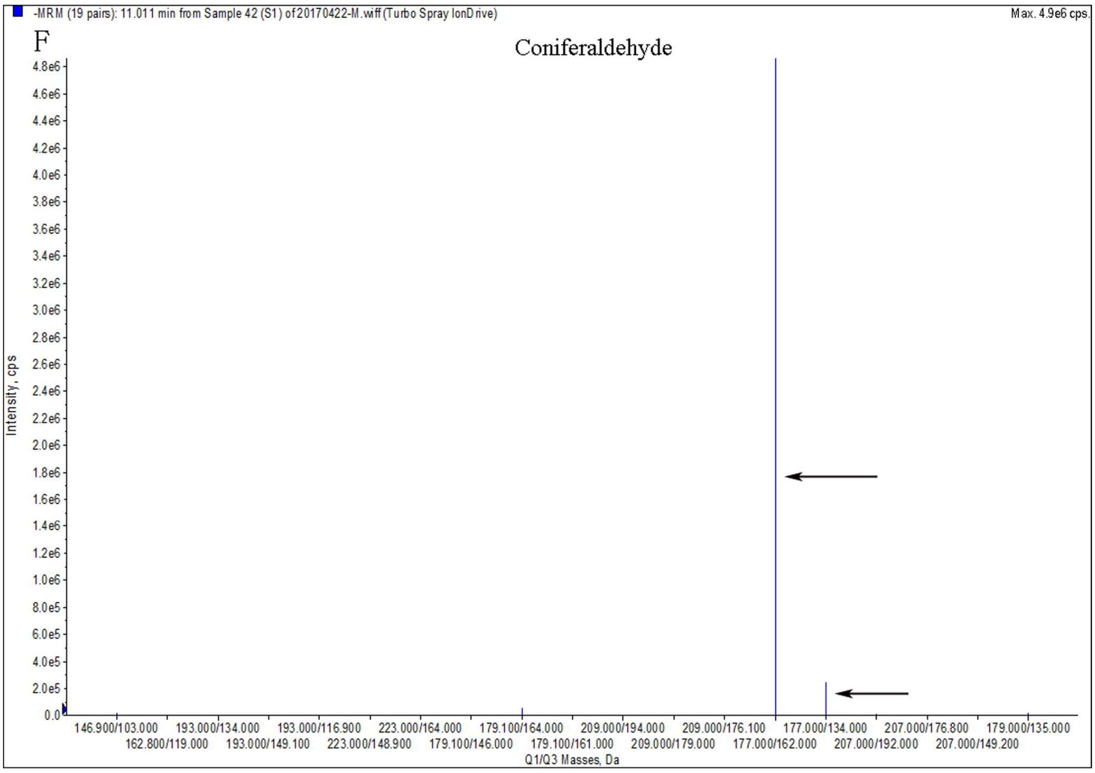
-MRM (19 pairs): 11.011 min from Sample 42 (S1) of 20170422-M.wiff (Turbo Spray IonDrive)
Max. 4.9e6 cps.
F
Coniferaldehyde
Intensity, cps
4.8e6
4.6e6
4.4e6
4.2e6
4.0e6
3.8e6
3.6e6
3.4e6
3.2e6
3.0e6
2.8e6
2.6e6
2.4e6
2.2e6
2.0e6
1.8e6
1.6e6
1.4e6
1.2e6
1.0e6
8.0e5
6.0e5
4.0e5
2.0e5
0.0
146.900/103.000
162.800/119.000
193.000/134.000
193.000/149.100
193.000/116.900
223.000/148.900
223.000/164.000
179.100/146.000
179.100/164.000
179.100/161.000
209.000/194.000
209.000/179.000
209.000/176.100
177.000/162.000
177.000/134.000
207.000/192.000
207.000/176.800
207.000/149.200
179.000/135.000
Q1/Q3 Masses, Da

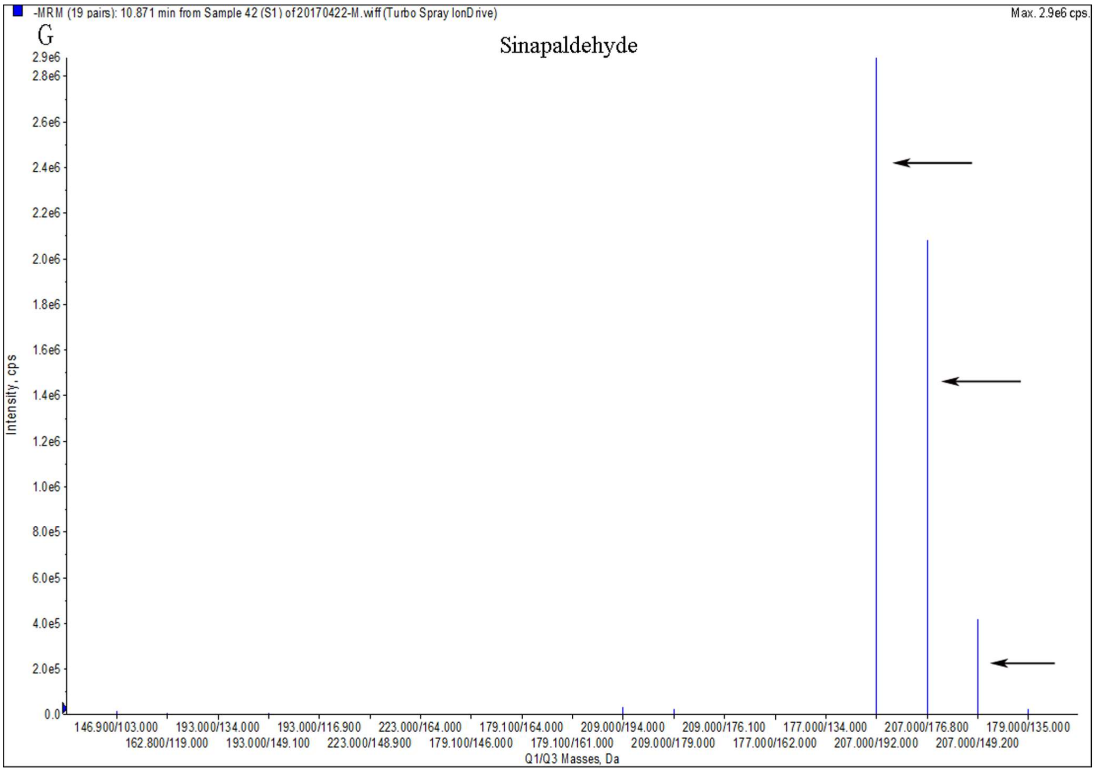
-MRM (19 pairs): 10.871 min from Sample 42 (S1) of 20170422-M.wiff (Turbo Spray IonDrive)
Max. 2.9e6 cps.
G
Sinapaldehyde
Intensity, cps
Q1/Q3 Masses, Da
146.900/103.000
162.800/119.000
193.000/134.000
193.000/149.100
193.000/116.900
223.000/148.900
223.000/164.000
179.100/146.000
179.100/164.000
179.100/161.000
209.000/194.000
209.000/179.000
209.000/176.100
177.000/162.000
177.000/134.000
207.000/192.000
207.000/176.800
207.000/149.200
179.000/135.000

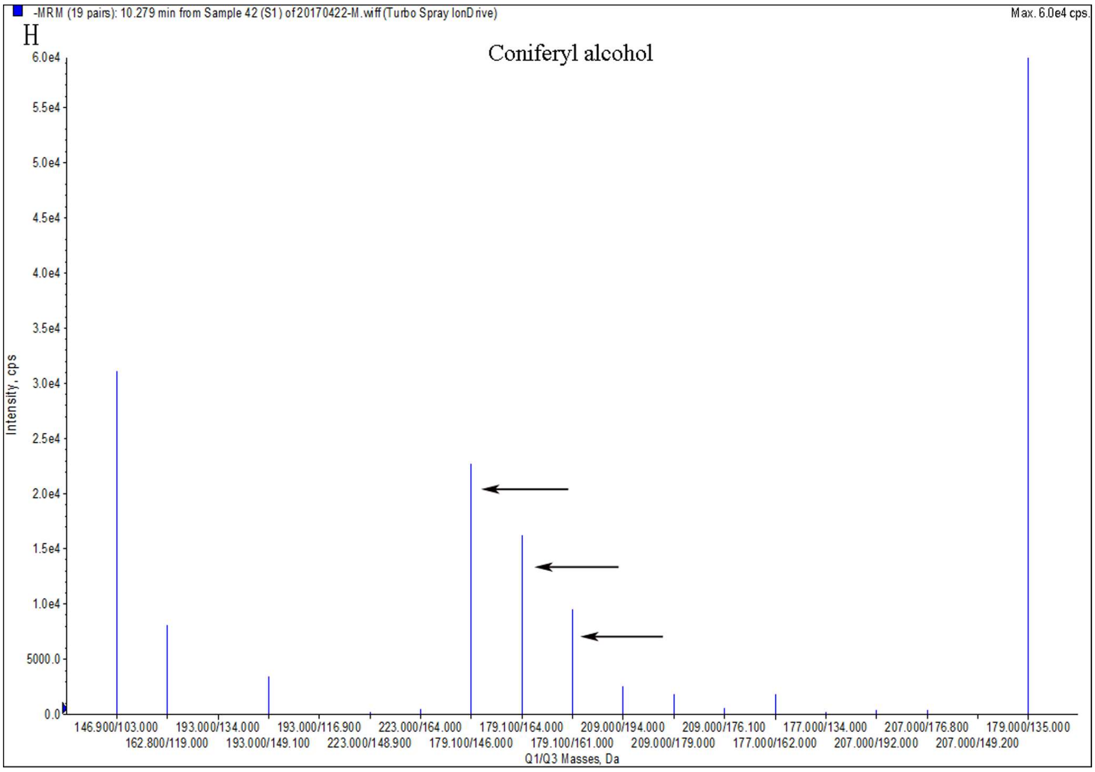
-MRM (19 pairs): 10.279 min from Sample 42 (S1) of 20170422-M.wiff (Turbo Spray IonDrive)
Max. 6.0e4 cps.
H
Coniferyl alcohol
Intensity, cps
Q1/Q3 Masses, Da
146.900/103.000
162.800/119.000
193.000/134.000
193.000/149.100
193.000/116.900
223.000/148.900
223.000/164.000
179.100/146.000
179.100/164.000
179.100/161.000
209.000/194.000
209.000/179.000
209.000/176.100
177.000/162.000
177.000/134.000
207.000/192.000
207.000/176.800
207.000/149.200
179.000/135.000

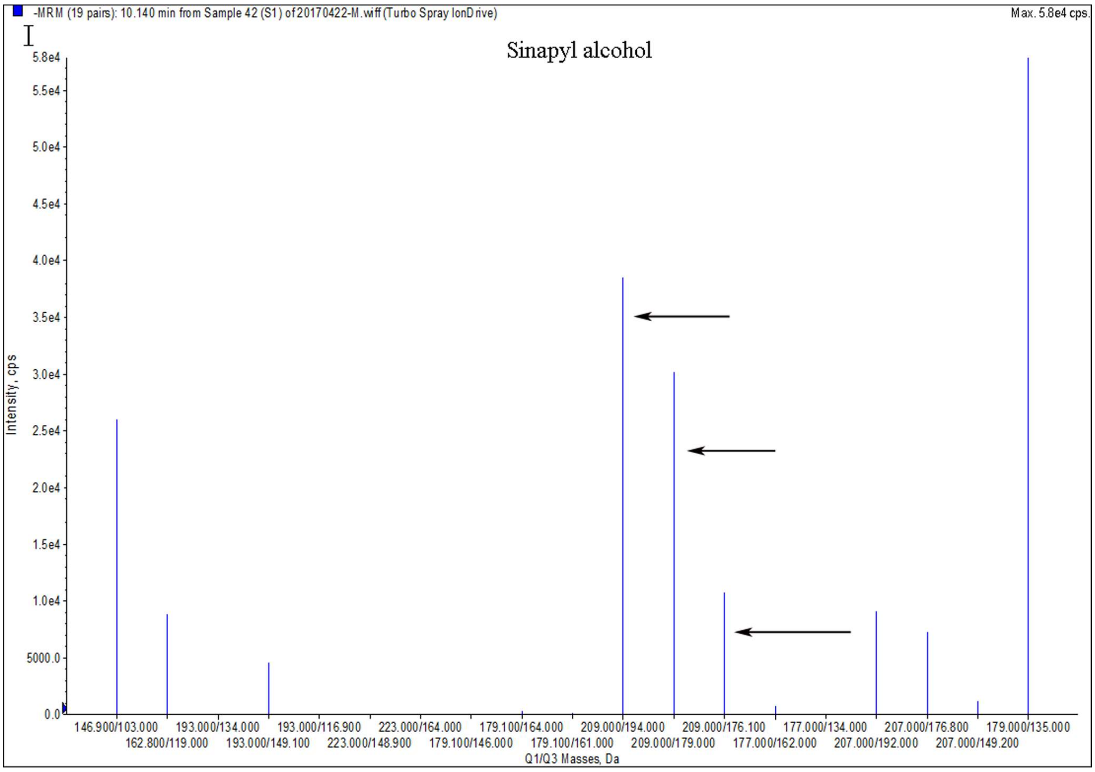
-MRM (19 pairs): 10.140 min from Sample 42 (S1) of 20170422-M.wiff (Turbo Spray IonDrive)
Max. 5.8e4 cps.
I
Sinapyl alcohol
Intensity, cps
Q1/Q3 Masses, Da
146.900/103.000
162.800/119.000
193.000/134.000
193.000/149.100
193.000/116.900
223.000/148.900
223.000/164.000
179.100/146.000
179.100/164.000
179.100/161.000
209.000/194.000
209.000/179.000
209.000/176.100
177.000/162.000
177.000/134.000
207.000/192.000
207.000/176.800
207.000/149.200
179.000/135.000

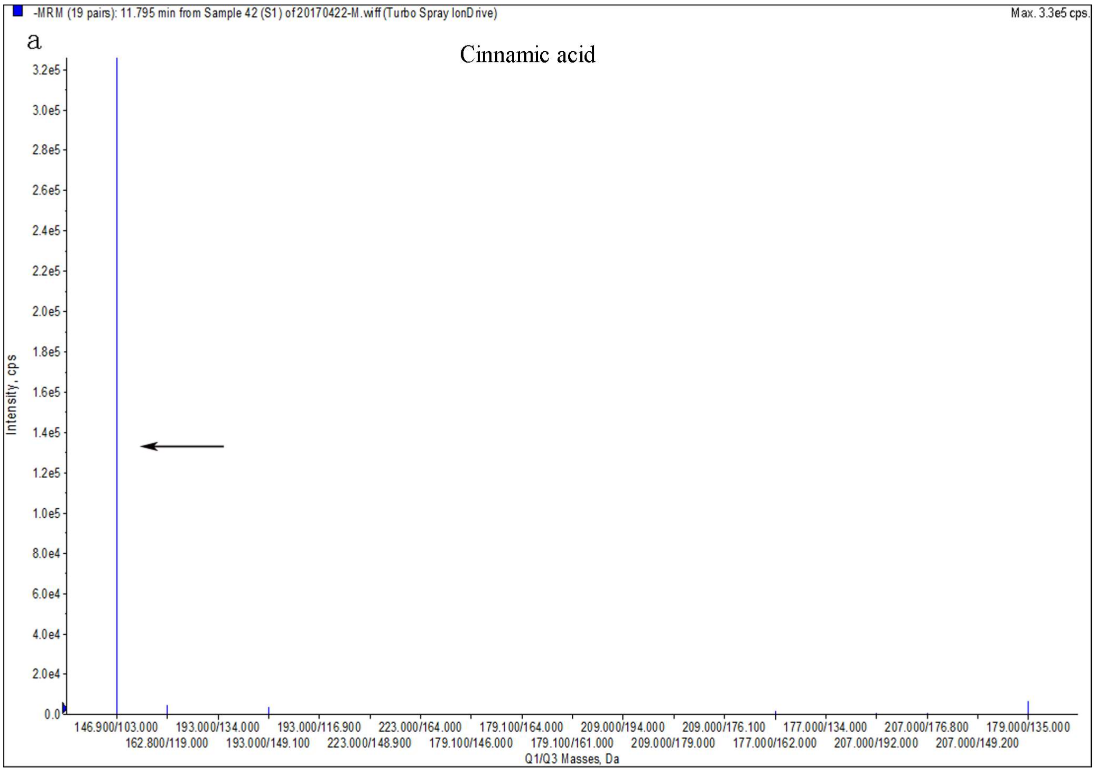
-MRM (19 pairs): 11.795 min from Sample 42 (S1) of 20170422-M.wiff (Turbo Spray IonDrive)
Max. 3.3e5 cps.
a
Cinnamic acid
Intensity, cps
Q1/Q3 Masses, Da

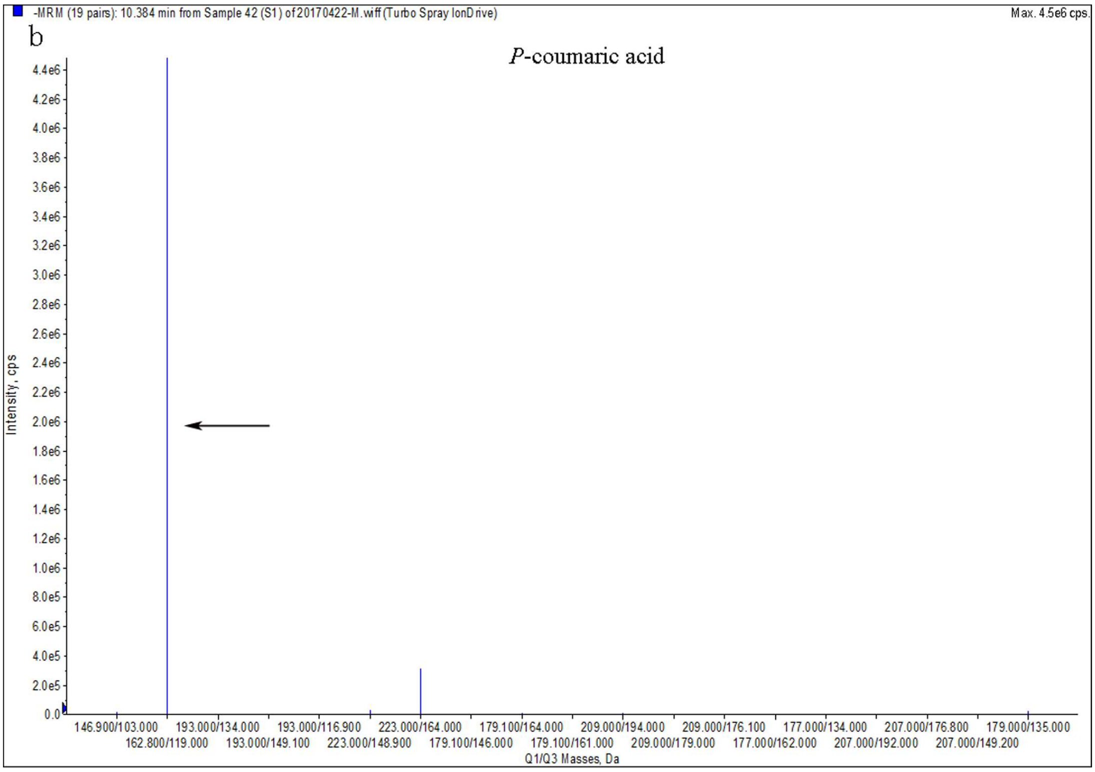

-MRM (19 pairs): 10.384 min from Sample 42 (S1) of 20170422-M.wiff (Turbo Spray IonDrive)
Max. 4.5e6 cps.
b
P-coumaric acid
Intensity, cps
Q1/Q3 Masses, Da

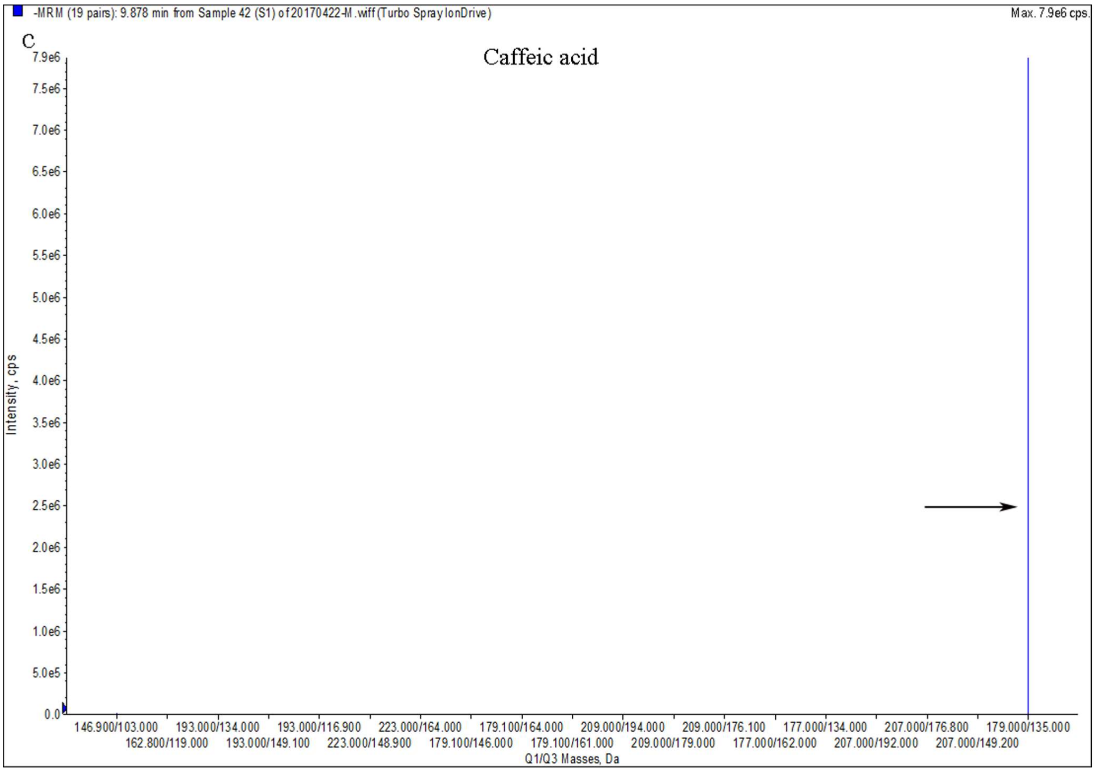

-MRM (19 pairs): 9.878 min from Sample 42 (S1) of 20170422-M.wiff (Turbo Spray IonDrive)
Max. 7.9e6 cps.
c
Caffeic acid
Intensity, cps
Q1/Q3 Masses, Da

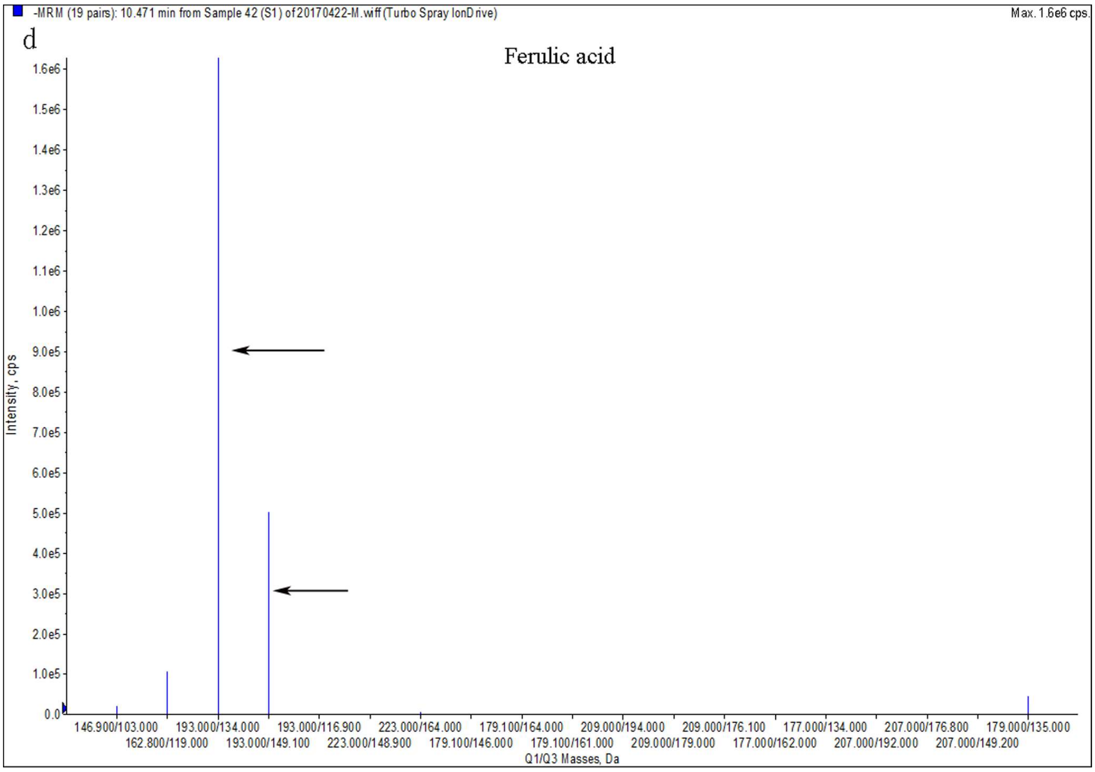
-MRM (19 pairs): 10.471 min from Sample 42 (S1) of 20170422-M.wiff (Turbo Spray IonDrive)
Max. 1.6e6 cps.
d
Ferulic acid
Intensity, cps
146.900/103.000
162.800/119.000
193.000/134.000
193.000/149.100
193.000/116.900
223.000/148.900
223.000/164.000
179.100/146.000
179.100/164.000
179.100/161.000
209.000/194.000
209.000/179.000
209.000/176.100
177.000/162.000
177.000/134.000
207.000/192.000
207.000/176.800
207.000/149.200
179.000/135.000
Q1/Q3 Masses, Da

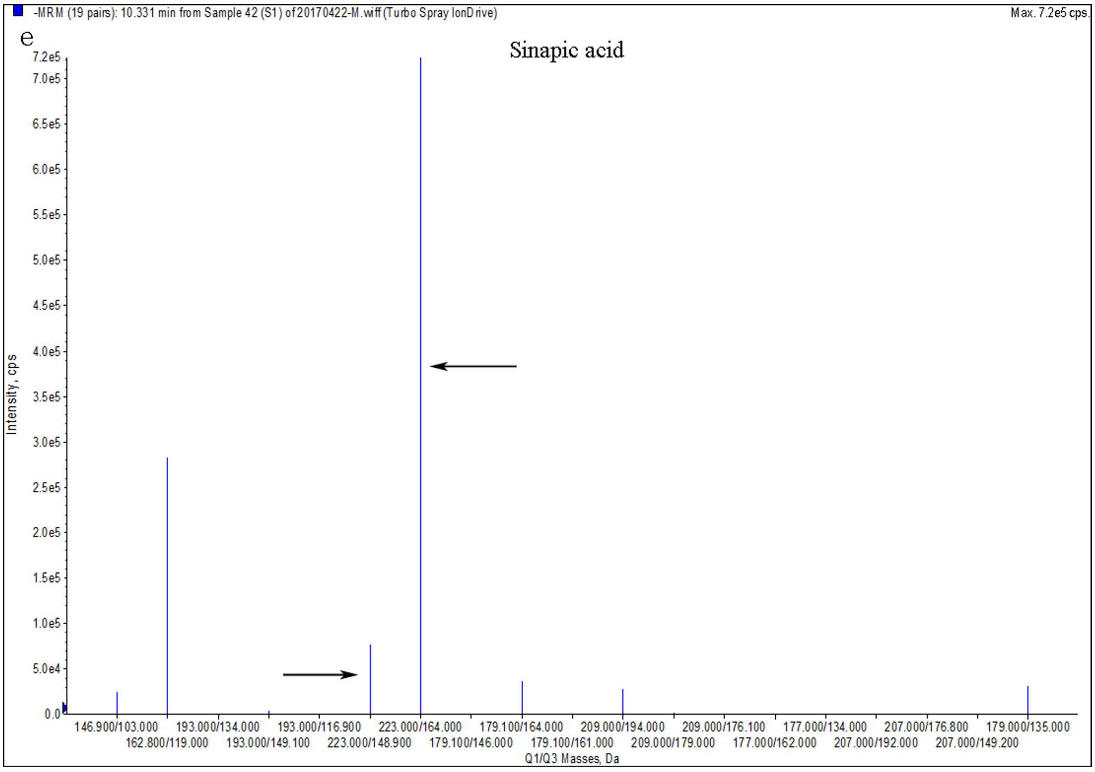
-MRM (19 pairs): 10.331 min from Sample 42 (S1) of 20170422-M.wiff (Turbo Spray IonDrive)
Max. 7.2e5 cps.
e
Sinapic acid
Intensity, cps
146.900/103.000
162.800/119.000
193.000/134.000
193.000/149.100
193.000/116.900
223.000/148.900
223.000/164.000
179.100/146.000
179.100/164.000
179.100/161.000
209.000/194.000
209.000/179.000
209.000/176.100
177.000/162.000
177.000/134.000
207.000/192.000
207.000/176.800
207.000/149.200
179.000/135.000
Q1/Q3 Masses, Da

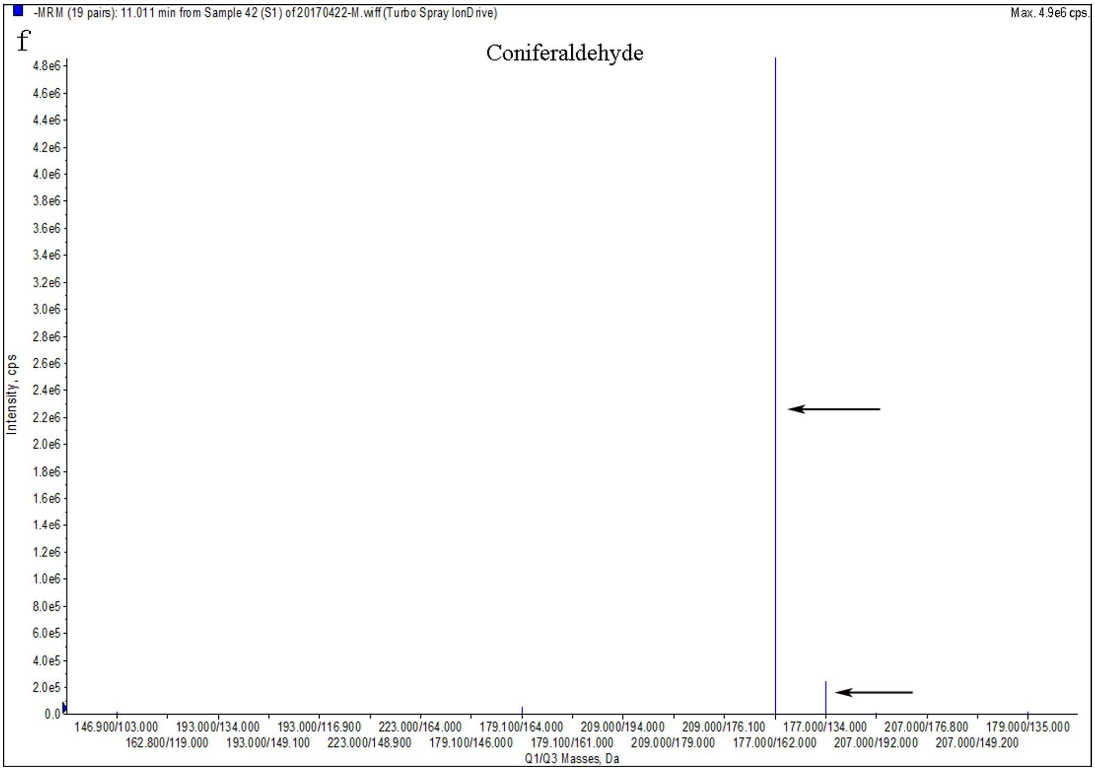
-MRM (19 pairs): 11.011 min from Sample 42 (S1) of 20170422-M.wiff (Turbo Spray IonDrive)
Max. 4.9e6 cps.
f
Coniferaldehyde
Intensity, cps
Q1/Q3 Masses, Da
146.900/103.000
162.800/119.000
193.000/134.000
193.000/149.100
193.000/116.900
223.000/148.900
223.000/164.000
179.100/146.000
179.100/164.000
179.100/161.000
209.000/194.000
209.000/179.000
209.000/176.100
177.000/162.000
177.000/134.000
207.000/192.000
207.000/176.800
207.000/149.200
179.000/135.000

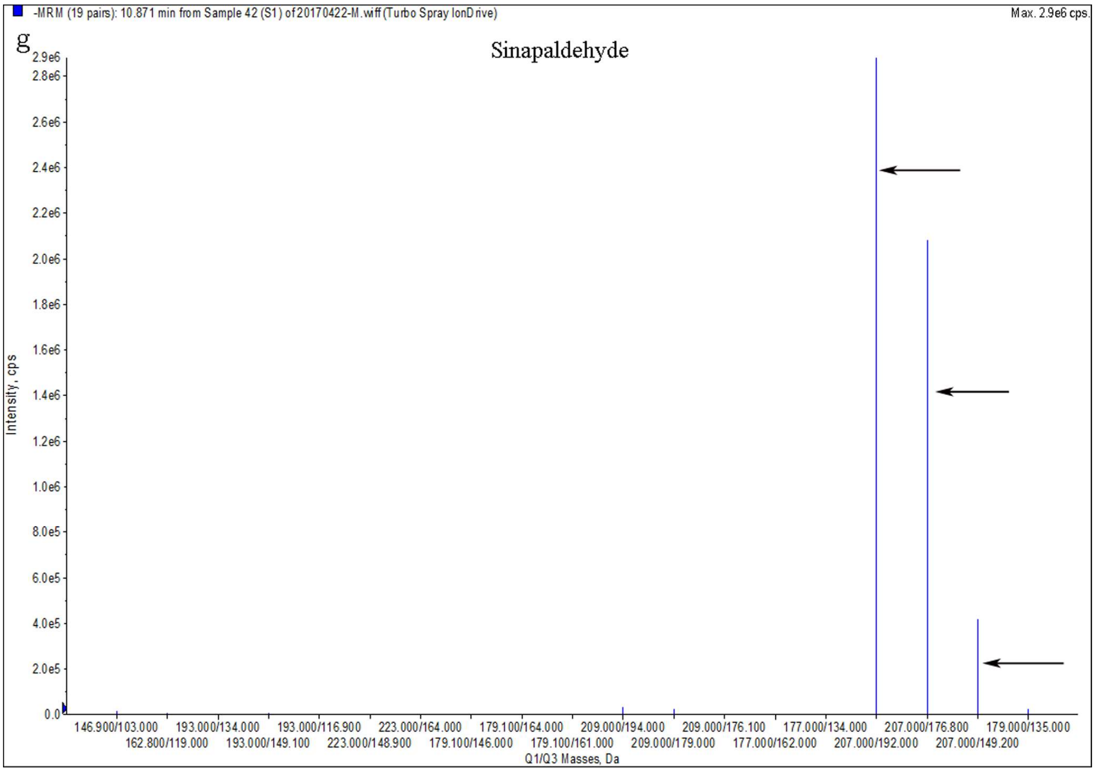
-MRM (19 pairs): 10.871 min from Sample 42 (S1) of 20170422-M.wiff (Turbo Spray IonDrive)
Max. 2.9e6 cps.
g
Sinapaldehyde
Intensity, cps
Q1/Q3 Masses, Da
146.900/103.000
162.800/119.000
193.000/134.000
193.000/149.100
193.000/116.900
223.000/148.900
223.000/164.000
179.100/146.000
179.100/164.000
179.100/161.000
209.000/194.000
209.000/179.000
209.000/176.100
177.000/162.000
177.000/134.000
207.000/192.000
207.000/176.800
207.000/149.200
179.000/135.000

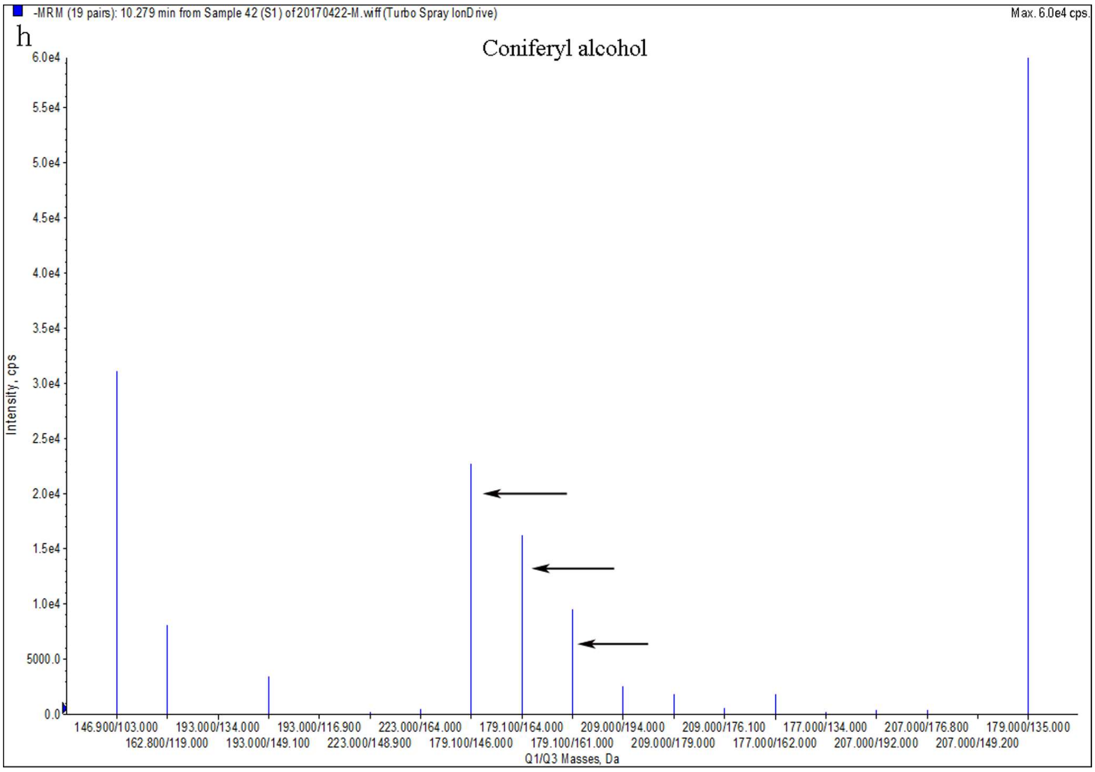

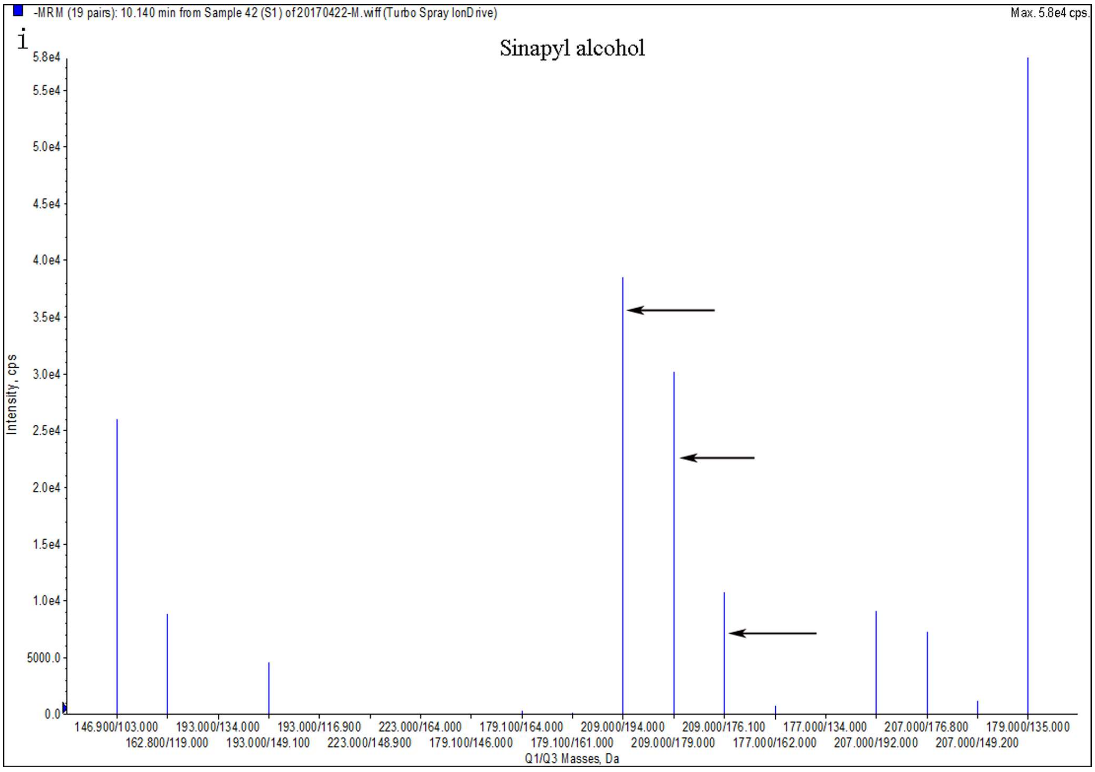

Figure S2 Sharacteristic ion pair spectrum of lignin synthesis intermediate metabolites in pear fruit

Note A-I: standards; a-i: samples;

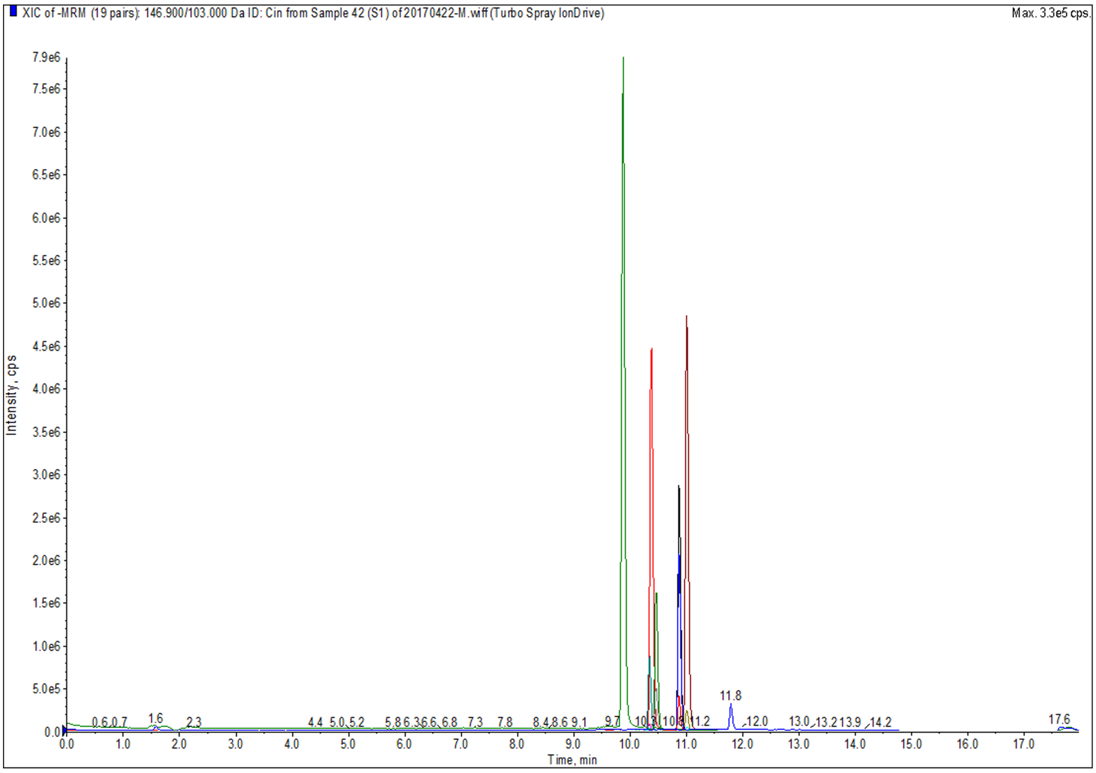

Figure S3 The total ion chromatogram of standards

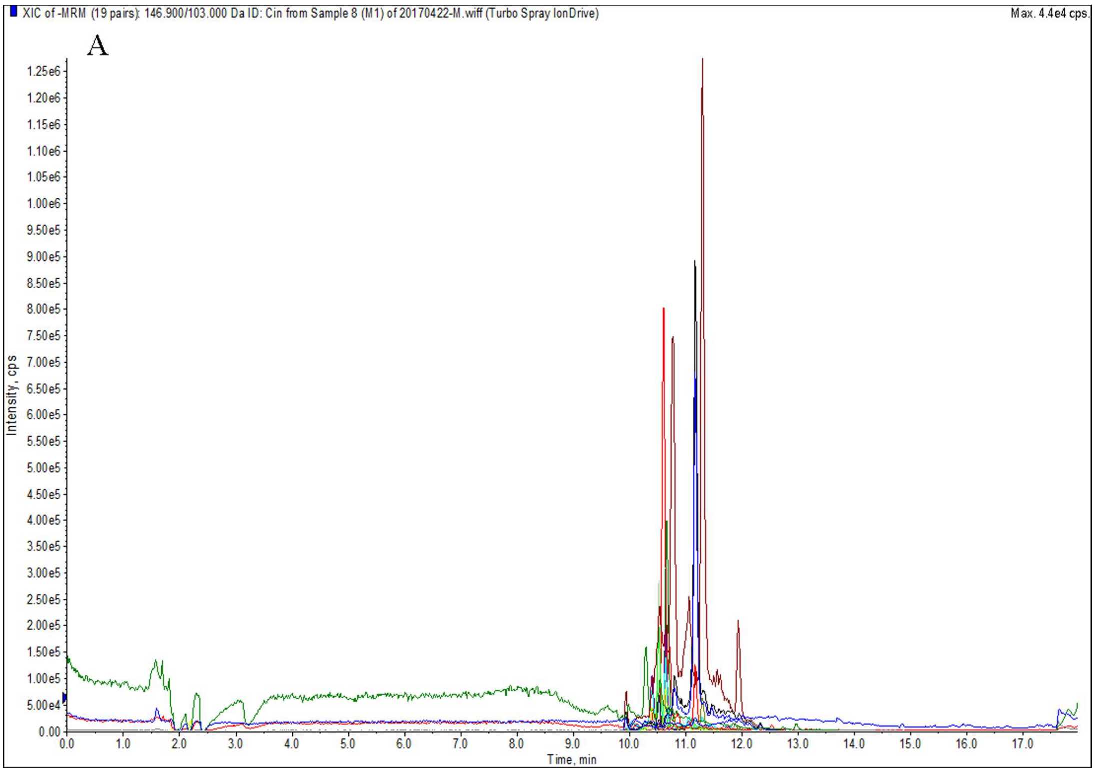

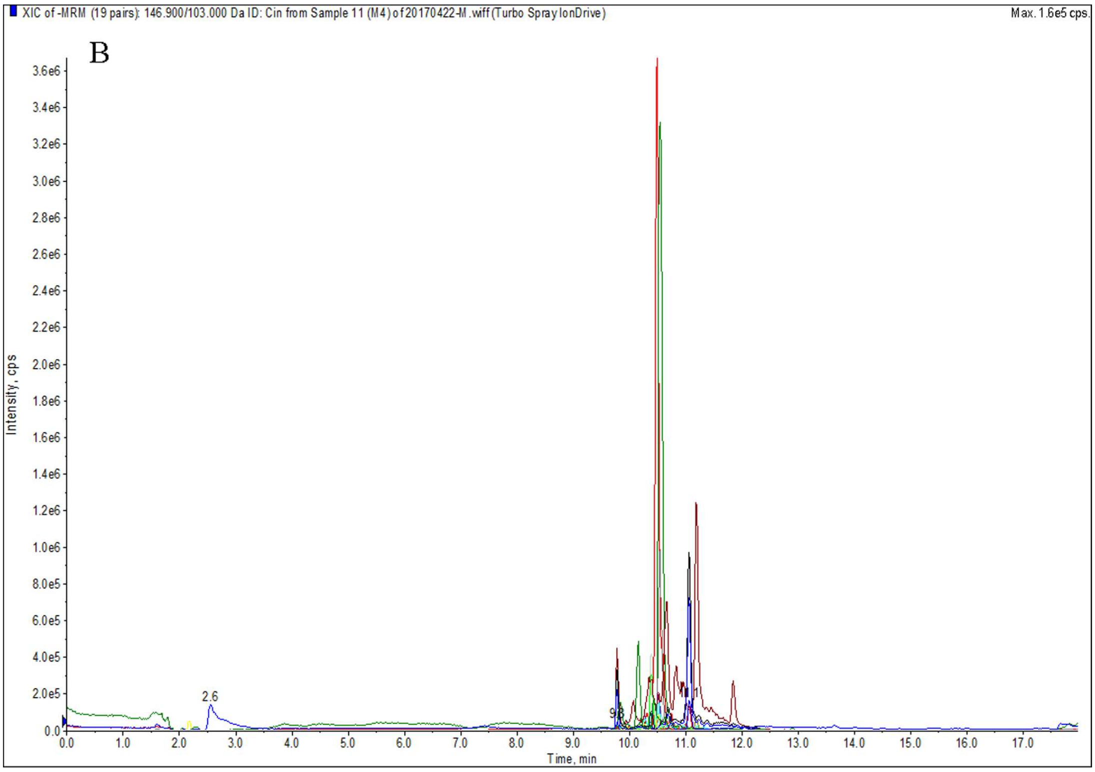
XIC of -MRM (19 pairs): 146.900/103.000 Da ID: Cin from Sample 11 (M4) of 20170422-M.wiff (Turbo Spray IonDrive)
Max. 1.6e5 cps.
B
Intensity, cps
Time, min
2.6

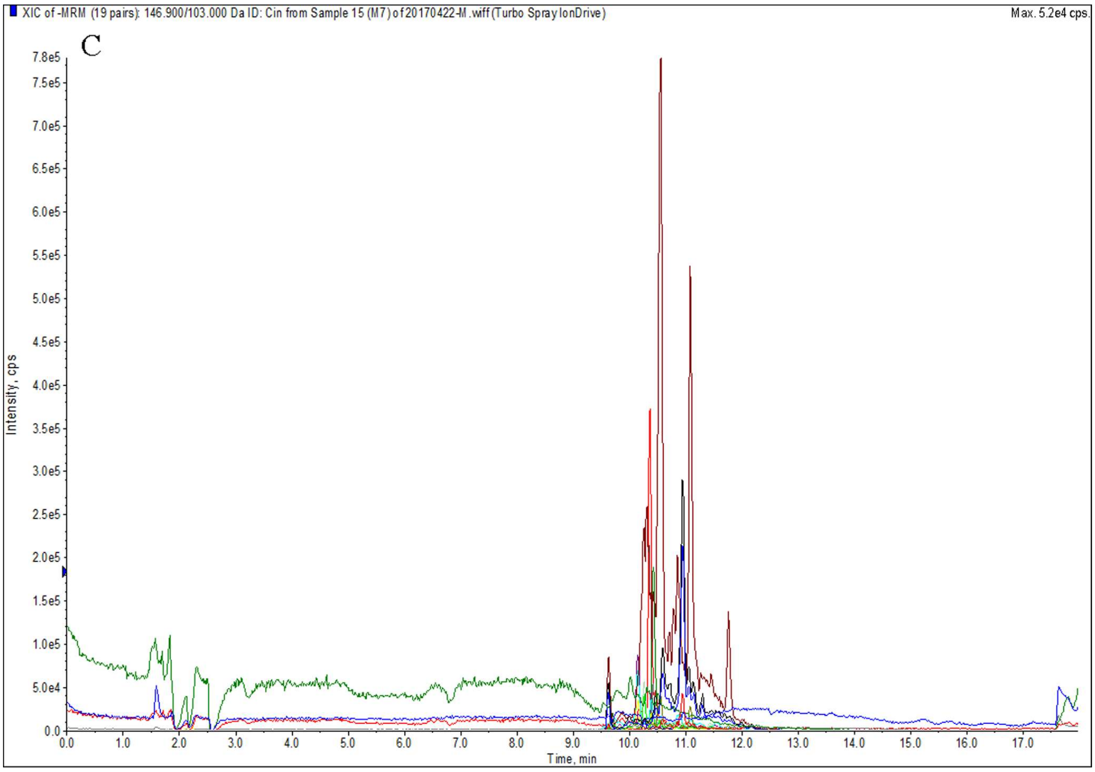
XIC of -MRM (19 pairs): 146.900/103.000 Da ID: Cin from Sample 15 (M7) of 20170422-M.wiff (Turbo Spray IonDrive)
Max. 5.2e4 cps.
C
Intensity, cps
Time, min

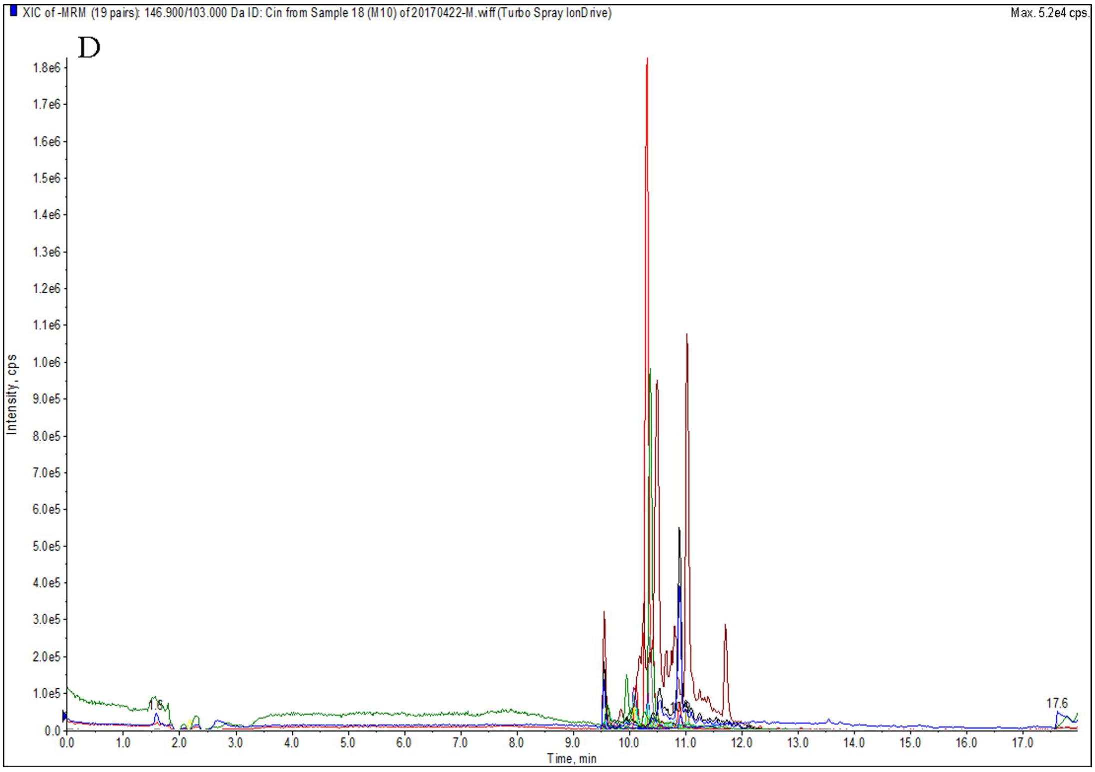

Figure S4 The total ion chromatogram of samples

Note A: DW (47 DAPs); B: DJ (47 DAPs); C: DW (63 DAPs); D: DJ (63 DAPs)
